# Identifying RNA Modifications by Direct RNA Sequencing Reveals Complexity of Epitranscriptomic Dynamics in Rice

**DOI:** 10.1016/j.gpb.2023.02.002

**Published:** 2023-02-11

**Authors:** Feng Yu, Huanhuan Qi, Li Gao, Sen Luo, Rebecca Njeri Damaris, Yinggen Ke, Wenhua Wu, Pingfang Yang

**Affiliations:** State Key Laboratory of Biocatalysis and Enzyme Engineering, School of Life Sciences, Hubei University, Wuhan 430062, China

**Keywords:** Direct RNA sequencing, Polyadenylated transcriptome, *N*^6^-methyladenosine, *N*^5^-methylcytosine, Rice

## Abstract

Transcriptome analysis based on high-throughput sequencing of a cDNA library has been widely applied to functional genomic studies. However, the cDNA dependence of most RNA sequencing techniques constrains their ability to detect base modifications on RNA, which is an important element for the post-transcriptional regulation of gene expression. To comprehensively profile the ***N***^**6**^**-methyladenosine** (m^6^A) and ***N***^**5**^**-methylcytosine** (m^5^C) modifications on RNA, **direct RNA sequencing** (DRS) using the latest Oxford Nanopore Technology was applied to analyze the transcriptome of six tissues in **rice**. Approximately 94 million reads were generated, with an average length ranging from 619 nt to 1013 nt, and a total of 45,707 transcripts across 34,763 genes were detected. Expression profiles of transcripts at the isoform level were quantified among tissues. Transcriptome-wide mapping of m^6^A and m^5^C demonstrated that both modifications exhibited tissue-specific characteristics. The transcripts with m^6^A modifications tended to be modified by m^5^C, and the transcripts with modifications presented higher expression levels along with shorter poly(A) tails than transcripts without modifications, suggesting the complexity of gene expression regulation. Gene Ontology analysis demonstrated that m^6^A- and m^5^C-modified transcripts were involved in central metabolic pathways related to the life cycle, with modifications on the target genes selected in a tissue-specific manner. Furthermore, most modified sites were located within quantitative trait loci that control important agronomic traits, highlighting the value of cloning functional loci. The results provide new insights into the expression regulation complexity and data resource of the transcriptome and epitranscriptome, improving our understanding of the rice genome.

## Introduction

Gene expression includes two major stages, transcription and translation, with the former generating RNAs and the latter generating proteins, which are spatially separated in eukaryote cells. Studies have shown the importance of post-transcriptional activities that occur involving mRNAs, including splicing, editing, capping, poly(A) tailing, and modification [1], [2], [3]. Compared with studies on the function of alternative splicing [4], [5] and poly(A) tail of mRNA [6], [7], [8], [9], [10], studies on the base modifications of RNA are still far behind, although they were first discovered more than 60 years ago [11]. To date, more than 160 RNA base modifications with different biological functions have been detected [12], [13], [14], which are much more abundant than the modifications on DNA. These modifications allow more complexity in gene expression regulation at the post-transcriptional level. Among them, *N*^6^-methyladenosine (m^6^A) is one of the most common modifications in the transcriptome of eukaryotes and occurs in nearly all kinds of RNAs [15]. Studies in humans have identified the proteins involved in the methylation of adenosine, demethylation, and recognition of m^6^A, revealing that m^6^A is essential for gene expression, tumor formation, stem cell fate, animal development, and RNA metabolism [15]. Moreover, another RNA modification, *N*^5^-methylcytosine (m^5^C), is also found to have important biological functions [16], [17]. Undoubtedly, it is of great importance to systematically identify these modifications among transcriptomes.

Several approaches have been developed to detect m^6^A and m^5^C modifications, although some challenges remain. Most of the sequencing methods of m^6^A depend on an m^6^A-specific antibody, whereby methylated RNA immunoprecipitation sequencing (MeRIP-seq) can identify m^6^A peaks [18], while photo-crosslinking-assisted m^6^A sequencing (PA-m^6^A-seq), m^6^A cross-linking immunoprecipitation (m^6^A-CLIP), and m^6^A individual-nucleotide-resolution cross-linking and immunoprecipitation (miCLIP) can obtain the base resolution of m^6^A [19], [20], [21]. The antibody-independent m^6^A sequencing methods, MAZTER-seq and m^6^A-sensitive RNA-endoribonuclease-facilitated sequencing (m^6^A-REF-seq), are based on endoribonuclease [22], [23], and two chemical labeling methods, m^6^A-label-seq and FTO-assisted m^6^A-selective chemical labeling method (m^6^A-SEAL), have also been recently developed [24], [25]. However, the application of these methods may be limited because of the intrinsic bias of antibodies, motif preference of endoribonuclease, and labeling efficiency [26]. The bisulfite-based sequencing method has a single-base resolution, and it is widely applied to detect m^5^C, although it is insensitive when detecting m^5^C in low abundance [27], [28]. Similar to m^6^A, m^5^C-specific antibodies are also applied to detect m^5^C peaks in transcriptomes [16], [29]. Moreover, methyltransferase-dependent methods of m^5^C, 5-azacytidine-mediated RNA immunoprecipitation (Aza-IP), and miCLIP, are also developed to enrich the m^5^C-modified transcripts [30], [31]. Nonetheless, unconverted cytosines via bisulfite treatment and overexpression of methyltransferase may result in false-positive detection of m^5^C sites [26], [32], [33], [34]. In addition, parallel control experiments for most of these methods are needed, and unsuitable approaches based on next-generation sequencing (NGS) have been applied to detect more than two different modifications simultaneously.

The direct RNA sequencing (DRS) technique recently developed by Oxford Nanopore Technology (ONT) provides an alternative way to characterize the transcriptome, wherein different ionic currents in nanoscale pores are generated and employed to discriminate nucleosides [35], [36], [37]. DRS data have higher correlations with cDNA nanopore data and Illumina datasets, and they tend to cover full transcripts in a strand-specific manner [35]. Importantly, sequences from DRS retain modification information because reverse transcription and polymerase chain reaction (PCR) amplification are not required, promisingly detecting multiple types of modifications in one experiment. DRS has been successfully applied to quantify transcripts at the isoform level, as well as assess ploy(A) tail length and base modification of m^6^A and m^5^C in human, *Caenorhabditis elegans*, and *Arabidopsis* transcriptome studies [37], [38], [39], [40], [41], [42], displaying its potential power in clarifying the complex transcriptome.

Rice is not only the staple food for more than half of the world’s population, but also a model monocot for molecular genetics studies because of its compact genome among cereals. Its high-quality reference genome has dramatically facilitated functional genomics research [43], [44], [45]. A further understanding of the complexity of the rice transcriptome and epitranscriptome might be very helpful in obtaining deeper insights into the mechanism of rice development. Transgenic expression of human RNA demethylase FTO in rice was found to mediate m^6^A demethylation, as well as induce chromatin openness and transcriptional activation, causing an increment in grain yield and biomass [46]. Rice transgenic lines stimulated root meristem cell proliferation and tiller bud formation, as well as promoted stress tolerance, whereas they did not affect cell size, shoot meristem cell proliferation, root diameter, and plant height [46], implying that m^6^A modification differentially regulates the developmental processes. The rice m^6^A methyltransferase OsFIP is indispensable for male gametogenesis, and the *osfip* mutant showed an early degeneration of microspores and abnormal meiosis [47], whereas m^6^A-modified genes were considerably different in the callus and leaf of rice [48], further indicating the importance of m^6^A in tissue-specific development. Furthermore, an investigation of m^5^C methyltransferase, OsNSUN2, in rice demonstrated that the *osnsnu2* mutant displayed heat-hypersensitivity phenotypes, and heat stress enhanced the m^5^C modification of mRNAs involved in photosynthesis and detoxification [49]. These studies indicate that m^6^A and m^5^C modifications play essential roles in rice. In the present study, DRS was applied to sequence mRNAs from six different developmental tissues to characterize the transcriptome in rice, and the transcripts targeted by m^6^A and m^5^C were simultaneously detected, before clarifying their effects on gene expression and biological function. Our results presented here provide new insights into the post-transcriptional regulation of rice development.

## Results

### Profiling the dynamic transcriptome of rice through DRS

**Figure 1:**
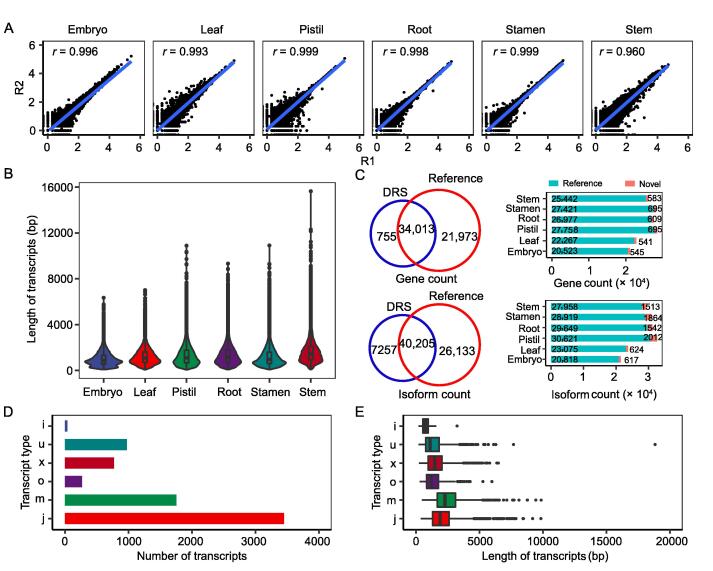
**Summary of DRS data for different rice tissues** **A.** Pearson correlation analysis between replicates of sequenced libraries in six rice tissues. *r* indicates the Pearson correlation coefficient. **B.** The length of transcripts detected by DRS in different tissues. **C.** The number of genes and isoforms identified by DRS and its comparison with the data in the reference genome (MSU7.0, https://rice.plantbiology.msu.edu/). **D.** The number of different types of novel transcripts identified by DRS through GffCompare pipeline analysis. Transcript type indicates the different types of novel transcripts: i, fully contained within a reference intron; j, multi-exon with at least one junction match; m, retained intron(s); o, other same strand overlapping with reference exons; u, none of the above (unknown, intergenic); x, exonic overlapping on the opposite strand. **E.** The length distribution of different types of novel transcripts. DRS, direct RNA sequencing.

To obtain a dynamic and comprehensive transcriptome of rice, the ONT DRS was applied to analyze different tissues, including the leaf, root, and stem from 2-week-old seedlings, the pistil and stamen from unopened floral buds, and the embryos from mature seeds (Figure S1). A total of 12 sequence libraries were constructed and loaded onto ONT R9.4 flow cells. Over 70 gigabyte bases and 94 million reads in all libraries were generated, and the read number of each sample ranged from 5.4 to 9.3 million (Table S1). The high Pearson correlation coefficient (*r*) between the two replicates of each tissue (Figure 1A) implied reproducible coverage. The read length distribution of six sequenced tissues was similar (Figure 1B), and the average read length for each sample ranged from 619 nt to 1013 nt, with the maximum read length being 15,373 nt and the average read quality score being more than 10 (Table S1), indicating high-quality DRS data.

Using stringTie [50] analysis, a total of 45,707 expressed transcripts corresponding to 34,768 genes were identified in the six tissues, with the numbers of expressed genes and transcripts ranging from 21,068 in the embryo to 28,453 in the pistil and from 21,435 in the embryo to 32,633 in the pistil, respectively (Figure 1C). Among them, 7257 novel isoforms that were not predicted in the reference genome were detected, and 755 novel genes that were not previously annotated were identified (Figure 1C; Table S2), of which 1756 novel transcripts that belong to intron retained might be immature transcripts. The largest numbers of novel isoforms and genes were identified in the pistil and stamen, respectively, whereas the lowest numbers of novel isoforms and genes were identified in the embryo of mature seed. The novel isoforms were divided into six categories according to GffCompare pipeline [51], including the following: i, fully contained within a reference intron; j, multi-exon with at least one junction match; m, retained intron(s); o, other same strand overlapping with reference exons; u, none of the above (unknown, intergenic); and  x, exonic overlapping on the opposite strand (Figure 1D). Different categories presented different distributions of transcript lengths (Figure 1E).

**Figure 2:**
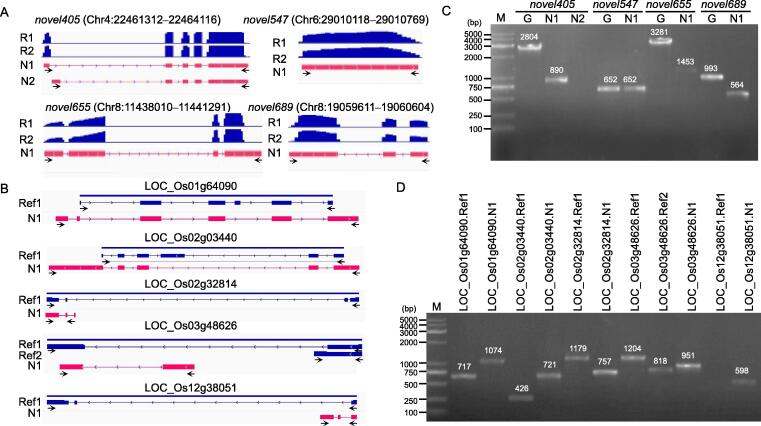
**Verification of the novel genes and transcripts identified by DRS** **A.** Novel genes that were not annotated in the reference genome. The read coverage of *novel405* and *novel547* was from the stem tissue, and the read coverage of *novel655* and *novel689* was from the pistil tissue. R1 and R2 represent the read coverage of independent biological replicates, and N1 and N2 represent the newly annotated transcripts. The arrows represent the location of primers. **B.** Novel transcripts that were different from the annotated genes in the reference genome. The blue color represents the annotated transcripts in the reference genome, and the red color represents the novel transcripts. Ref1 and Ref2 indicate different transcripts annotated in the reference genome. **C.** Verification of novel genes through RT-PCR. The same primer was used to amplify genomic DNA and cDNA. The cDNA template for *novel405*, *novel547*, *novel655*, and *novel689* was from the stem, stem, pistil, and pistil tissues, respectively. G represents the band amplified from genomic DNA; M represents marker bands. **D.** Verification of the novel isoforms through RT-PCR. The specific primer for each transcript was designed, and the cDNA template for LOC_Os01g64090, LOC_Os02g03440, LOC_Os02g32814, LOC_Os03g48626, and LOC_Os12g38051 was from the root, pistil, stem, stem, and root tissues, respectively. RT-PCR, reverse transcription-polymerase chain reaction.

To confirm the existence of the novel transcripts and genes, four novel genes (Figure 2A) and five novel isoforms (Figure 2B, Figure S2; Table S3) were subjected to PCR amplification and sequencing. The PCR band shifts in agarose gel were identical to the predicted length, whereas *novel405*.N2 and LOC_Os12g38051.N1 could not be efficiently amplified because of the low expression levels (Figure 2C and D, Figure S3). Alignment of the sequenced data (Table S4) with reference sequences also verified the accuracy of the predicted transcripts. These data indicate the reliability of the identified novel transcripts, which could be used for further analyses.

### DRS allowed identifying tissue-specific expression of genes and transcripts

**Figure 3:**
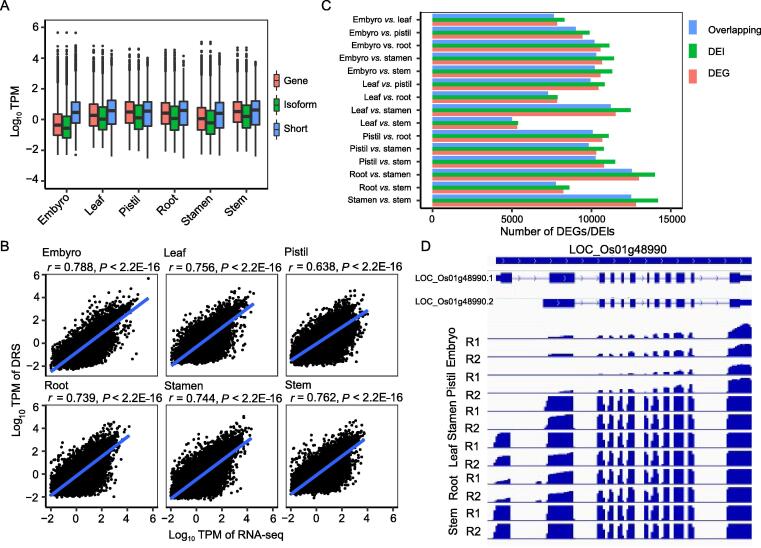
**Analysis of the expression of genes and isoforms detected in six tissues** **A.** The mRNA expression at gene and isoform levels. Gene indicates the expression at the gene level from the DRS data; isoform indicates the expression at the isoform level; short indicates the expression at the gene level from the short-read sequencing data of the cDNA library using Illumina platform. **B.** Pearson correlation analysis of expression at the gene level in six tissues determined by DRS and short-read sequencing from the cDNA library using Illumina platform. **C.** Analysis of the DEGs and DEIs among tissues. Overlapping represents the number of genes overpping between DEGs and genes presenting DEIs. **D.** The expression of LOC_Os01g48990 in an isoform-specific manner in the six tissues. The read coverage was displayed through IGV software. R1 and R2 represent two different replicates. TPM, transcripts per kilobase per million; DEG, differentially expressed gene; DEI, differentially expressed isoform; IGV, integrative genomics viewer.

The ONT DRS technique can directly sequence RNA, based on which transcripts with different isoforms can be distinguished, thus facilitating the quantification of mRNAs at the isoform level. Comparison among all the tissues showed that about 48.8% (16,955) of the genes were commonly expressed in all six tissues (Figure S4), whereas only 37.9% (18,495) of the isoforms were commonly expressed (Figure S5), indicating the tissue-specific expression of genes and their different isoforms. Further comparison showed that the median of gene expression quantified from short-read sequencing was higher than that from DRS, and the isoform expression level was lower (Figure 3A). Pearson correlation analysis was conducted between DRS and Illumina sequencing data to check the reliability of DRS on the quantification of gene expression. The notable correlation in all the six tissues (Figure 3B) verified the precision of DRS. The differentially expressed genes (DEGs) and differentially expressed isoforms (DEIs) of the six tissues were further identified using salmon tools at the gene and transcript levels, respectively [52], and a large number of DEGs and DEIs were discovered in each comparison (Figure 3C). The leaf *vs.* stem comparison revealed the lowest number of DEGs and DEIs, whereas the leaf *vs.* root and stem *vs.* root comparisons revealed the second and third lowest numbers of DEGs and DEIs, respectively (Figure 3C). In contrast, the stamen *vs.* root/stem/leaf comparisons revealed the largest numbers of DEGs and DEIs (Figure 3C). Generally, more than 95% of the DEIs had their corresponding genes identified as DEGs (Figure 3C, Figure S6). Some genes contained more than one transcript, whereby some were identified as DEIs with no observable changes at the gene expression level, while some genes were identified as DEGs without any of their transcripts being identified as DEIs (Figure S6), suggesting the existence of tissue-specific genes and transcripts. One gene, LOC_Os01g48990, which displayed tissue-specific expression of its transcripts, was randomly selected to verify these results. The read coverage showed that LOC_Os01g48990.1 was expressed in the leaf, root, and stem, whereas LOC_Os01g48990.2 was expressed in the embryo, pistil, and stamen (Figure 3D). Moreover, the number of DEGs from DRS data was lower than in Illumina sequencing, whereas about 85% of DEGs detected in DRS were also identified in Illumina sequencing (Figure S7).

### High repeatability of m^6^A and m^5^C identification through DRS

As a new technique, DRS has an advantage in identifying modifications [35]. The development of Tombo software makes it feasible to detect these modified sites [53]. Two modifications, m^6^A and m^5^C, were identified in six tissues with two replicates in the present study. Because of the lower accuracy of DRS compared with NGS, the repeatability of m^6^A and m^5^C identification was evaluated. About 63% to 78% of m^6^A-modified sites were simultaneously detected, and over 90% of m^6^A-modified genes in most tissues were identified in both replicates (Figure S8A and B), whereas the fraction (frequency of modified sites in the transcript) of overlapping sites in two replicates was significantly highly correlated (Figure S8C). Similar results were also found in m^5^C-modified sites and genes (Figure S9), indicating that the sites detected in both replicates have good repeatability, and that independent biological replicates are necessary. The repeatedly detected sites were thus subjected to further analysis. To evaluate the reliability of modifications identified by DRS, the m^6^A MeRIP data from Nipponbare root samples of 15-day-old seedlings [46] were compared with DRS data of root samples (Figure S10). The results demonstrated that over 50% of m^6^A-modified genomic regions contained m^6^A sites identified by DRS, and about 70% of m^6^A-modified genes detected by MeRIP were also identified by DRS, implying the reliability of DRS data.

### The m^6^A and m^5^C modifications on transcripts occurred in a common or tissue-specific manner

**Figure 4:**
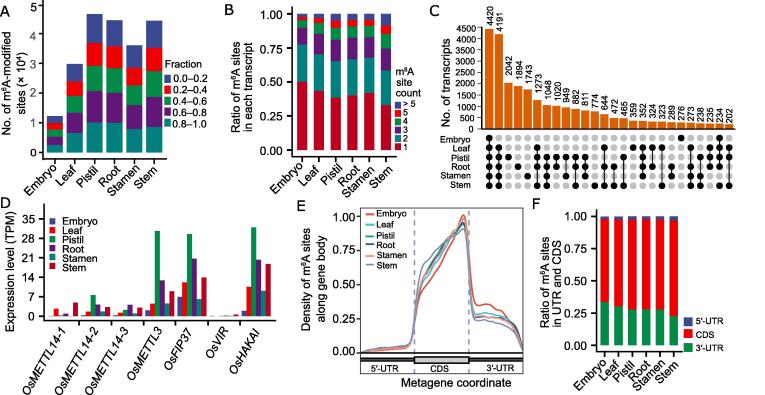
**Profiling of m^6^A modification in the transcripts of rice tissues** **A.** The number of m^6^A-modified sites. Each site was classified into different categories on the basis of its fraction. **B.** The ratio of transcripts with a different number of m^6^A-modified sites. **C.** The number of commonly-detected and tissue-specific m^6^A-modified transcripts. **D.** The expression levels of possible m^6^A writer genes. *OsMETTL14-1*, *OsMETTL14-2*, and *OsMETTL14-3* are homologs of human *METTL14*; *OsMETTL3* is a homolog of human *METTL3*; *OsFIP37* is a homolog of *AtFIP37*; *OsVIR* is a homolog of *AtVIR*; *OsHAKAI* is a homolog of *AtHAKAI*. **E.** The density of m^6^A-modified bases along the gene body in six tissues. The position of each modified site along the gene body was normalized by the length of the transcript using R pipeline MetaPlotR. **F.** The ratio of m^6^A-modified sites distributed in the 5′-UTR, CDS, and 3′-UTR. m^6^A, *N*^6^-methyladenosine; UTR, untranslated region; CDS, coding sequence.

**Figure 5:**
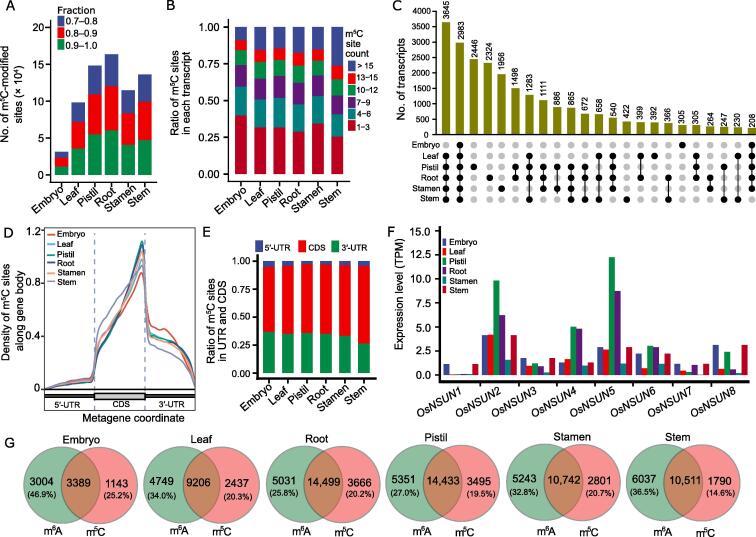
**Profiling of m^5^C modification in the transcripts of rice tissues** **A.** The number of m^5^C-modified sites in different tissues. Each site was classified into different categories on the basis of its fraction. **B.** The ratio of transcripts with a different number of m^5^C-modified sites. **C.** The number of commonly-detected and tissue-specific m^5^C-modified transcripts. **D.** The density of m^5^C-modified bases along the gene body. The position of each modified site along the gene body was normalized by the length of the transcript using R pipeline MetaPlotR. **E.** The ratio of m^5^C-modified sites distributed in the 5′-UTR, CDS, and 3′-UTR in the six tissues. **F.** The expression levels of possible m^5^C methyltransferase genes. *OsNSUN1*–*OsNSUN8* correspond to LOC_Os08g0484400, LOC_Os09g0471900, LOC_Os02g0320100, LOC_Os02g0724600, LOC_Os09g0551300, LOC_Os08g0365900, LOC_Os02g0217800, and LOC_Os09g0477900, respectively. **G.** The comparison of m^5^C- and m^6^A-modified transcripts in each tissue. m^5^C, 5-methylcytosine.

m^6^A is the most prevalent post-transcriptional modification, and it is necessary for regulating gene expression [54]. A total of 81,722 m^6^A-modified sites located within 28,059 transcripts were identified in the whole genome, with the number of sites in each tissue ranging from 12,271 in the embryo to 46,535 in the pistil (Table S5). The site numbers in the root and stem were slightly lower than those in the stem, whereas the site numbers in the leaf and stamen were 2–3-fold greater than those in the embryo, with more than half of these sites having a fraction over 0.5 (Figure 4A). The average number of m^6^A sites in each transcript ranged from 1.92 in the embryo to 2.67 in the stem, and the number of genes with m^6^A modification ranged from 5152 in the embryo to 14,051 in the pistil (Table S5). Most of the transcripts had less than three m^6^A sites, whereas over 25% of isoforms in the stem had more than four m^6^A sites (Figure 4B). The fraction of transcripts with more than six modified sites displayed a wide variation (from 0 to 1), but the maximum fraction in these transcripts (median value > 0.92) was significantly higher than that in all modified transcripts (median value < 0.75) (Figure S11). Considering the variable number of m^6^A modifications among different tissues, the intersection of transcripts with m^6^A modification was analyzed. A small number of transcripts (*n* = 4420) overlapped in all tissues, with 4191 transcripts commonly presented in the leaf, pistil, root, stamen, and stem (Figure 4C). Moreover, a proportion of isoforms displayed tissue-specific modification by m^6^A, including 2042 in the pistil, 1894 in the root, 1743 in the stamen, 774 in the stem, 359 in the leaf, and 276 in the embryo (Figure 4C). To clarify whether the m^6^A methylase affects the status of m^6^A modification in each tissue, the expression levels of eight putative m^6^A methylase genes were analyzed. Except for *OsMTC*, other genes were expressed in all tissues, with *OsMETTL3*, *OsFIP37*, and *OsHAKAI* showing relatively high expression levels (Figure 4D). Consistent with the m^6^A intensity in each tissue, most of these genes had higher expression levels in the pistil, root, and stem, with the lowest expression levels observed in the embryo (Figure 4D). These sites were distributed from the 5′-untranslated region (5′-UTRs) to 3′-UTR, mainly around the stop codon of the coding sequence (CDS) (Figure 4E). There was an apparent shift of the site distribution toward the 5′-UTR in the stem (Figure 4E), in which the largest number of transcripts containing multiple m^6^A modifications was identified. Approximately 40% of m^6^A-modified sites presented the GGACA motif, whereas the other three types of motifs (AGACT, GGACC, and GGACT) also had a considerable ratio (Figure S12).

m^5^C is another popular internal RNA modification. A total of 338,907 sites with m^5^C modifications located within 25,869 transcripts were identified, with the m^5^C sites in each tissue ranging from 31,339 in the embryo to 163,430 in the root, in which the fraction of most sites was more than 0.8 (Figure 5A; Table S6). The average m^5^C site number per isoform was 6.9 in the embryo and 11.1 in the stem, whereas the other four tissues featured ∼ 8.5 m^5^C sites, exceeding the number recorded for m^6^A modification. Most of the transcripts had more than four m^5^C sites, and over 25% of transcripts in the stem had more than 15 m^5^C sites (Figure 5B). Among these transcripts with more than 15 m^5^C sites, the fraction of each site ranged from 0.7 to 1.0, and the maximum fraction in these transcripts was significantly higher than in all the detected transcripts (Figure S13). The number of m^5^C-modified transcripts commonly identified in the six tissues was 2983 (Figure 5C). The peak of m^5^C modification was located around the start codon and stop codon, and the CDS region had the higher proportion of m^5^C sites in all tissues (Figure 5D). Similar to m^6^A modification, there was also a shift toward the 5′-UTR in the stem (Figure 5E). The expression of eight putative m^5^C methyltransferase genes [49] was also checked. Most had high expression among all six tissues (Figure 5F). Specifically, the expression of two genes, *OsNSUN2* and *OsNSUN5*, was much higher in the pistil and root than in the other four tissues (Figure 5F). Although the lowest expression of methyltransferase genes was presented in the stamen (Figure 5F), the number of m^5^C-modified sites was not the lowest. Nine bases around the modified C were analyzed for conserved elements, with (A/T)GC(T/A) being the most representative element covering 96,434 sites, whereas the other three potential elements were (A/C)(A/T)CAX(C/A)(T/A) (where X = A/T/C/G), TC(A/G/C)(G/A)(G/T), and CAG(A/G)CT (Figure S14).

Because over half of the expressed transcripts were either m^6^A- or m^5^C-modified, it was necessary to check if the transcript was co-targeted by m^6^A and m^5^C. A comparison of the transcripts modified with m^6^A and m^5^C in each tissue showed that more than half of the m^6^A-modified transcripts were also modified by m^5^C, and over 75% of the m^5^C-modified transcripts were also modified by m^6^A in the rice transcriptome (Figure 5G). The number of co-modified transcripts varied in different tissues and ranged from 3389 in the embryo to 14,499 in the root (Figure 5G). Moreover, approximately 20% of the transcripts of these modified genes were not modified by m^6^A or m^5^C in each tissue (Figure S15), implying the isoform-specific patterns of both modifications.

### Both m^6^A and m^5^C modifications were correlated with the expression level and length of poly(A) tail of transcripts

**Figure 6:**
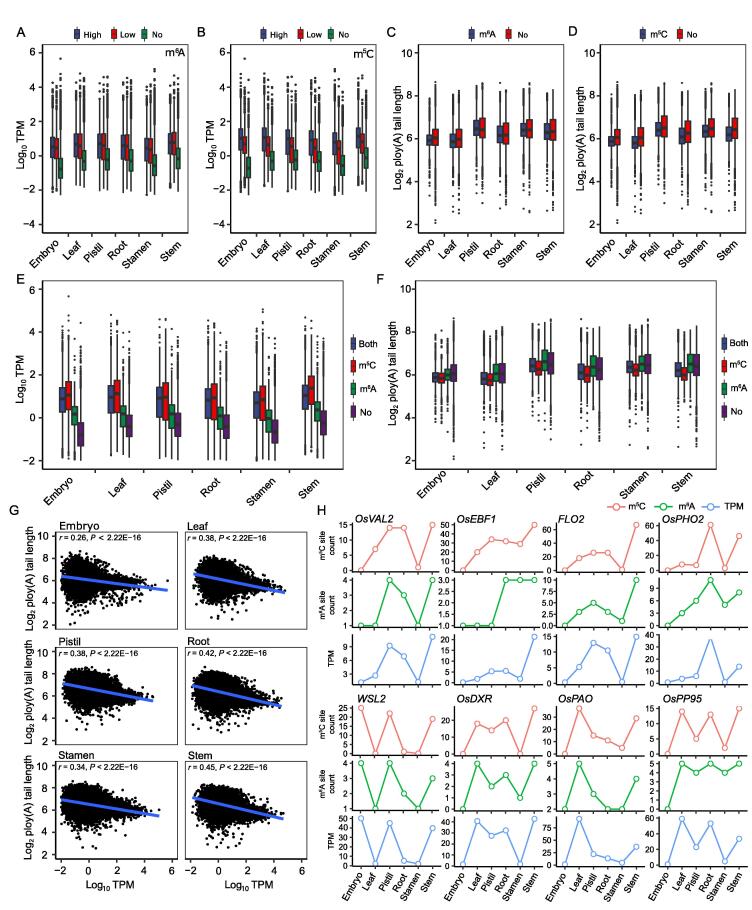
**Relationship of m^6^A and m^5^C with transcript expression level and poly(A) tail length** **A.** Comparison of the expression level of transcripts with and without m^6^A modification in each tissue. High indicates the transcripts with the maximum fraction ranging from 0.5 to 1.0; low indicates the transcripts with the maximum fraction ranging from 0.0 to 0.5; no indicates the transcripts without m^6^A modification. **B.** Comparison of the expression level of transcripts with and without m^5^C modification in each tissue. High indicates the transcripts with the maximum fraction ranging from 0.9 to 1.0; low indicates the transcripts with the maximum fraction ranging from 0.7 to 0.9; no indicates the transcripts without m^5^C modification. **C.** Comparison of poly(A) tail length of transcripts with and without m^6^A modification in each tissue. m^6^A indicates the transcripts modified by m^6^A; no indicates the transcripts not modified by m^6^A. **D.** Comparison of poly(A) tail length of transcripts with and without m^5^C modification in each tissue. m^5^C indicates the transcripts modified by m^5^C; no indicates the transcripts not modified by m^5^C. **E.** The expression level of transcripts with different modifications. Both indicates the transcripts modified by both m^6^A and m^5^C; m^6^A indicates the transcripts modified by m^6^A only; m^5^C indicates the transcripts modified by m^5^C only; no indicates the transcripts without m^6^A and m^5^C modifications. **F.** The poly(A) tail length of transcripts with different modifications. **G.** Pearson correlation analysis between poly(A) tail length and expression level of isoforms. **H.** Comparison of expression level with the numbers of m^6^A and m^5^C sites among six tissues in eight cloned genes: *OsVAL2* (LOC_Os07g48200.1), *OsEBF1* (LOC_Os06g40360.1), *FLO2* (LOC_Os04g55230.1), *OsPHO2* (LOC_Os05g48390.1), *WSL5* (LOC_Os03g04660.1), *OsDXR* (LOC_Os01g01710.1), *OsPAO* (LOC_Os03g05310.1), and *OsPP95* (LOC_Os07g32380.1). ***, *P* < 0.001 for each comparison.

To understand the function of m^6^A and m^5^C, we analyzed the correlation between these two modifications and the expression levels of their targeted transcripts. The results demonstrated that m^6^A- or m^5^C-modified transcripts had significantly higher expression levels than the transcripts with no modification, whereas transcripts with higher fractions of modification sites also had higher expression levels (Figure 6A and B). The transcripts with more m^6^A or m^5^C sites tended to have higher expression levels (Figures S16 and S17), which was apparent for m^5^C. To determine the potential interaction of other factors with transcript expression, we analyzed the relationship between the modifications and poly(A) tail length of the corresponding transcripts. It was found that transcripts with either m^6^A or m^5^C modification had significantly shorter poly(A) tail length than those without modification (Figure 6C and D). Although the number of m^6^A modification sites seemed to have no effect on the length of the poly(A) tail (Figure S18), the number of m^5^C sites had a negative relationship with the length of the poly(A) tail (Figure S19). To identify any additive effects between m^6^A and m^5^C, the expression of transcripts with both modifications was compared with that with or without either modification. Although transcripts with both modifications had relatively higher expression levels than those with only m^6^A modifications or without modifications (Figure 6E), they were similar to those only modified by m^5^C (Figure 6E). These results indicate that there is no obviously additive effect on promoting the expression of transcripts, with m^5^C being more effective. Their impact on poly(A) tail length was contrasted with their impact on the expression (Figure 6F). According to these results, it seems that poly(A) tail length is negatively correlated with transcript expression. To verify this assumption, the relationship between the poly(A) tail length and the transcript abundance was analyzed. Consistently, the poly(A) tail length was negatively related to the abundance of transcripts in all the tissues (Figure 6G).

The proportion of m^6^A- or m^5^C-modified sites located in the 5′-UTR, CDS, and 3′-UTR in each transcript was further calculated, and the correlation between the modification location and the expression level or poly(A) tail length of transcripts was analyzed (Table S7). The results demonstrated that the m^6^A or m^5^C modification sites located in the 5′-UTR and CDS were weakly positively correlated with the expression level. In contrast, the modifications located in the 3′-UTR were weakly negatively correlated with the expression level. A contrasting tendency was identified in the comparison between m^5^C location and poly(A) tail length, implying that the sites modified by m^6^A or m^5^C in the 5′-UTR and CDS may have been correlated with the expression level and poly(A) tail length of transcripts. To further verify the relationship between the expression level and the numbers of m^5^C and m^6^A sites, eight genes (*OsVAL2*, *OsEBF1*, *FLO2*, *OsPHO2*, *WSL5*, *OsDXR*, *OsPAO*, and *OsPP95*) showing differential modifications among the six tissues were selected to check their expression levels and transcript modification statuses. The results showed that genes with higher expression levels also had more modified m^5^C and m^6^A sites (Figure 6H, Figure S20), implying that m^5^C and m^6^A modifications do correlate with their expression.

### The modified transcripts were involved in central metabolic pathways and exhibited tissue-specific characteristics

**Figure 7:**
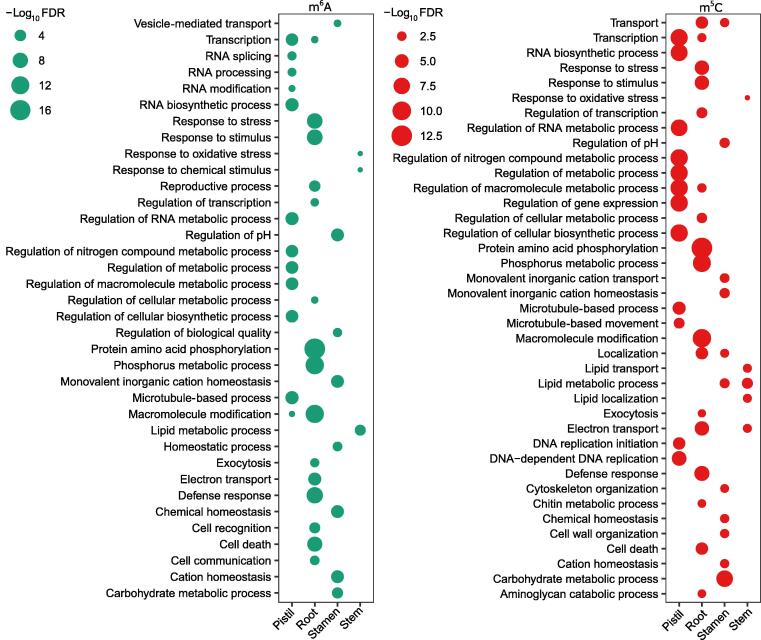
**GO analysis of tissue-specific transcripts with m**^**6**^**A and m**^**5**^**C modifications in each tissue through agriGO** The left panel shows the enriched GO terms of tissue-specific transcripts modified by m^6^A in each tissue, and the right panel shows the enriched GO terms of tissue-specific transcripts modified by m^5^C in each tissue. The enriched GO terms were selected according to FDR < 0.05. GO, Gene Ontology; FDR, false discovery rate.

Since both modifications could affect the abundance of their target transcripts, we wanted to determine if there were any selections on the target genes, especially in different tissues. Gene Ontology (GO) analysis was conducted on the transcripts modified by m^6^A and/or m^5^C. The transcripts with either m^6^A or m^5^C modifications overlapped in all tissues, and they were mainly involved in translation, different kinds of metabolic processes, gene expression, protein-related processes, and transport (Figure S21), indicating that both modifications might affect central life activities. GO enrichment analysis also provided some clues on the functions of these tissue-specific transcripts with m^6^A and m^5^C modifications (Figure 7). The pistil-specific transcripts with m^6^A modification were mainly involved in RNA metabolism including biosynthesis, splicing, processing, and modification, whereas some of the pistil-specific transcripts with m^5^C modification were enriched in the DNA replication process (Figure 7). Root-specific m^6^A- and m^5^C-modified transcripts were mainly involved in protein phosphorylation, phosphorus metabolism, macromolecule modification, cell communication and recognition, and stress and stimulus responses (Figure 7). Stamen-specific m^6^A- and m^5^C-modified transcripts were mainly involved in the pH, ion, and chemical homeostatic regulation process, whereas some of the transcripts with m^5^C modification were enriched in cell wall and cytoskeleton organization as well as lipid and carbohydrate metabolism (Figure 7). Interestingly, lipid metabolic process-related transcripts with m^6^A and m^5^C were particularly enriched in the stem (Figure 7). These results showed that transcripts modified with m^6^A and m^5^C were involved in similar functions, indicating an association between m^6^A and m^5^C modifications. We further analyzed the potential functions of transcripts that were commonly or specifically modified by m^6^A and m^5^C in each tissue. GO analysis of transcripts that were commonly modified by m^6^A and m^5^C revealed enrichment in multiple biological processes such as localization, metabolic, regulation, and transport in all tissues, whereas some GO terms were enriched in specific tissues such as translational initiation and elongation in the embryo, DNA repair and response to DNA damage stimulus in the pistil, cell homeostasis in the leaf, and purine nucleotide-related metabolic processes in the stamen (Figure S22). A few GO terms were simultaneously enriched in transcripts that were explicitly modified by m^6^A or m^5^C in each tissue, and most GO terms presented tissue and modification specificity (Figure S23), implying the differential functions of transcripts with m^6^A or m^5^C modifications. These data collectively demonstrate the similar and differential functions of m^6^A- or m^5^C-modified transcripts in a tissue-specific manner.

### Most genes with m^6^A and m^5^C modifications were located within quantitative trait loci

**Figure 8:**
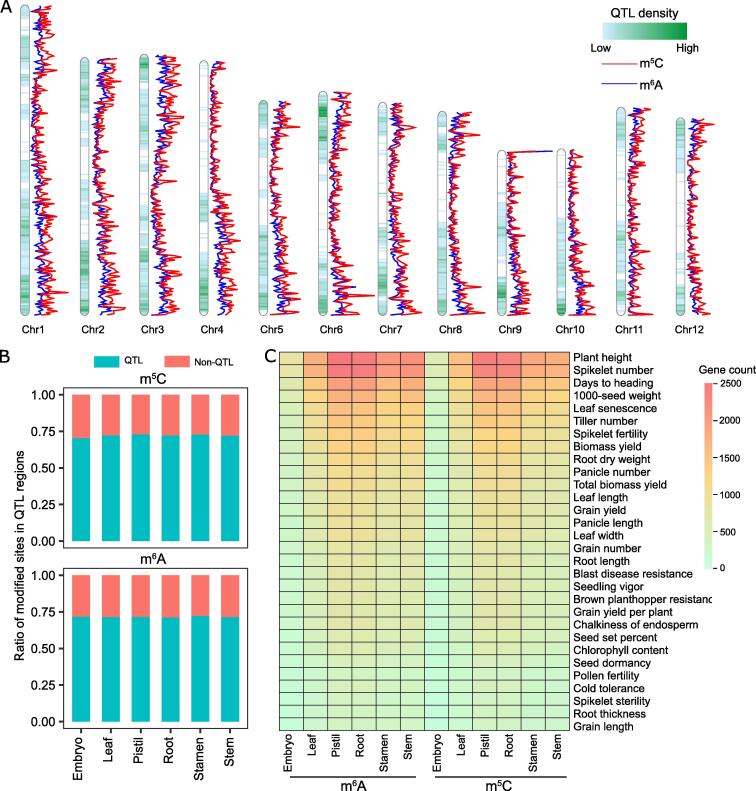
**ComparisonofRNA base-modified regions withpreviously mapped QTLs** **A.** The distribution of QTLs, m^5^C sites, and m^6^A sites along the chromosome with a 200-kb window size. The QTL information was downloaded from Gramene (https://www.gramene.org), and QTL intervals no more than 2 Mb were selected for further analysis. The display was drawn using the R package “RIdeogram”. **B.** The ratio of m^5^C- and m^6^A-modified sites localized within QTL regions. **C.** Heatmap showing the number of RNA base-modified genes localized within the QTL regions. The top 30 traits are shown. QTL, quantitative trait locus.

To further characterize whether m^6^A- and m^5^C-modified transcripts could affect any important agronomy traits, we analyzed the distribution of genes encoding the m^6^A- and m^5^C-modified transcripts, and we compared them with previously identified quantitative trait loci (QTLs) along the chromosomes with 200-kb windows. The results showed that m^5^C and m^6^A sites had similar distribution along the chromosome, and the regions with higher QTL density tended to have higher RNA base modifications (Figure 8A). Approximately 75% of the sites modified with m^5^C and m^6^A in each tissue were mapped in QTL regions (Figure 8B). This result suggests that m^5^C and m^6^A modification might play important roles in regulating the expression of genes located in or close to QTLs. Moreover, over 200 genes modified by m^6^A or m^5^C were located within QTL regions associated with 30 agronomy traits, which involved multiple processes, including development, yield, fertility, flowering, and biotic and abiotic stress (Figure 8C), implying that these genes with modified RNA bases may determine the important agronomy traits in the rice genome.

## Discussion

In the last two decades, noteworthy achievements have been realized in rice genomic research, greatly facilitating genetic and breeding studies. However, it is still elusive how the genome is concordantly expressed to realize its function. Hence, dissecting the dynamic combination of gene expression products or intermediates will be very important to uncover the mechanism of rice development and environmental response. Among relevant methods, transcriptome analysis is a critical approach to dissecting the transcripts, which is highly dependent on high-throughput sequencing techniques [55]. It has been established that only dissecting the transcripts is not enough to characterize their function. Many post-transcriptional activities involve mRNAs, which might be very important in regulating gene expression [1], [2], [3]. However, because of the limitations of the canonical RNA sequencing (RNA-seq) technique, these post-transcriptional activities cannot be finely characterized. The newly developed method DRS has been demonstrated to have an outstanding ability to concurrently identify these activities in humans, yeast, *C. elegans*, and *Arabidopsis* [35], [37], [41], [42]. Here, DRS was applied to characterize the transcriptome of six developmental tissues of rice. About 0.2% and 2% of the detected genes and isoforms were identified as novel genes and isoforms, respectively (Figure 1C), indicating that DRS could help identify more isoforms. Characterization and verification of the novel genes and isoforms, especially their tissue-specific expression patterns, could help improve the annotation of the rice genome and obtain new information on their functions. However, the DRS data only covered 60%–61% of the isoforms and genes annotated in the reference genome (Figure 1C). More than 22% (9776) of expressed genes, especially those with low transcripts per kilobase per million (TPM < 1), detected in RNA-seq could not be detected by DRS. This indicates that DRS may not be powerful enough to detect low-abundance transcripts, which is consistent with its characteristic of not amplifying the targets. It might be necessary to combine DRS and canonical RNA-seq techniques to comprehensively explore the transcriptome complexity, accurately quantify the transcripts, and expand the number of genes and isoforms in a tissue-specific manner.

In addition to the advantages of transcript identification and isoform quantification, DRS can detect the base modifications of RNA, which supposedly play important regulatory roles at the post-transcriptional level. Over 160 types of RNA modifications have been discovered [12], among which m^6^A and m^5^C have been verified to play key roles in development and stress response [56]. Antibody-based high-throughput sequencing techniques have been successfully used for transcriptome-wide mapping of m^6^A and m^5^C modifications of RNA for many eukaryotes such as yeast [29], [57], *Arabidopsis* [16], [58], [59], rice [48], [49], and maize [60]. Accordingly, the dominantly conserved motifs for m^6^A RRACH (R = A/G; H = A/C/U) enriching near the stop codon and 3′-UTR [61], and those for m^5^C sites in the CDS and UTR with the conserved motifs HACCR (H = A/U/C; R = A/G) and CTYCTYC(Y = U/C) [16], [59] have been characterized. Because DRS can directly sequence RNA without reverse and amplification processes, it can more accurately detect the base modifications, as proven by recent studies [37], [40]. We globally mapped m^6^A and m^5^C modifications through DRS in developmental rice tissues. The distribution region and conserved motifs for m^6^A in this study were similar to previous reports [54] (Figure 4E and F, Figure S12). Although the distribution region for m^5^C modification was also consistent with previous results [16] (Figure 5D and E), new conserved motifs were identified in our study (Figure S14). Thus, further studies on other species are required to determine the species specificity of these findings. Moreover, a high proportion of isoforms with both modifications was detected (Figure 5G). However, we did not find any additive effects on the gene expression (Figure 6E). It would be interesting to know if isoforms with one of the modifications could facilitate other modifications. Furthermore, m^5^C- or m^6^A-modified genes displayed isoform-specific modifications (Figure S15), and modification sites located within 5′-UTR, CDS, and 3′-UTR had potentially differential effects on transcript expression (Table S7). These primary data hint at the importance of sequencing RNA molecules at the transcript level.

The biological importance of m^6^A and m^5^C has been confirmed by previous studies [15], [17]. These modifications can affect the stability or translation efficiency of target mRNAs. Until now, there is still very little direct evidence from any specific mRNAs. In this study, we found that transcripts containing both modifications displayed higher expression levels and shorter poly(A) tails than those without modification (Figure 6A and B), and this effect was dependent on the number of modification sites, especially for m^5^C (Figures S16–S19). Specifically, the m^6^A and m^5^C modification intensities of eight cloned genes were highly associated with their expression levels among different tissues (Figure 6E). Moreover, the fraction of m^6^A- or m^5^C-modified sites showed dramatic variations (Figure 4A and 5A). In contrast, transcripts with higher fractions tended to display high expression levels (Figure 6A and B). The transcripts with m^6^A sites that fell into > 5 categories or with m^5^C sites that fell into > 15 categories presented a significantly higher maximum fraction than all modified transcripts (Figure 4B and 5B, Figures S11 and S13), implying that the effect of modification on transcript expression was also fraction-dependent. These findings indicated that m^6^A and m^5^C might be able to promote the stability of their modified transcripts, with m^5^C being more effective. However, how these modifications correlate with the length of the poly(A) tail is still an open question, which includes the intrinsic factors of modifications and the association of the length of the poly(A) tail with the expression level of transcripts.

GO enrichment analysis showed that the modified transcripts are widely involved in all aspects of biological processes. However, there were some tissue-specific modified groups (Figure 7, Figures S21–S23). The occurrence of modification was seemingly related to a specific biological process or tissue development, which has also been shown in strawberry fruit development [62] and in the sexual reproduction of *Chlamydomonas reinhardtii* [63]. The selection of target genes seems to be a meaningful problem, which was also addressed in this study. Among all the detected transcripts, most of the modified isoforms were found to be located within mapped QTLs controlling important agronomical traits such as yield, flowering, stress, and fertility (Figure 8), indicating there might be selectivity toward the targets to be modified. This selection bias might be related to the biological function of modifications.

## Materials and methods

### Planting materials and sampling

Rice (*Oryza sativa* L. subsp. *japonica* cultivar Nipponbare) was grown in the field of Hubei University, Wuhan, China. Leaves, stems, and roots from the two-week-old seedlings were collected after germinating and growing in an artificial climate chamber under 28 °C/25 °C, 16-h/8-h light/dark conditions using a 1/2 Murashige and Skoog medium plate. The pistils and stamens were separated and collected from the booting stage in the field, and the embryos were peeled from the mature dry seeds. All tissues were frozen immediately in liquid nitrogen and stored at −80 °C for further use. Each sample was collected in duplicate.

### RNA extraction and isolation

The total RNA of each sample was extracted using Trizol reagent (Catalog No. 15596026, Invitrogen, Gaithersburg, MD) according to the manufacturer’s instructions; it was then precipitated with 2.5 M LiCl, and DNase I (Catalog No. M0303L, New England Biolabs, Ipswich, MA) was added to remove genomic DNA. The quality of RNA was detected using a NanoDrop One spectrophotometer (NanoDrop Technologies, Wilmington, DE) and Qubit 3.0 fluorometer (Life Technologies, Carlsbad, CA). A total of 30 μg of qualified RNA was utilized to enrich poly(A) RNA through the mRNA NEBNext poly(A) mRNA magnetic isolation module (Catalog No. E7490S, New England Biolabs) according to the manufacturer’s specifications.

### Library construction and sequencing

Poly(A) RNA (∼ 500 ng) was used for nanopore DRS. The DRS library was constructed according to the ONT SQK-RNA002 kit protocol, including the optional reverse transcription step recommended by ONT. The library was loaded onto ONT R9.4 flow cells and sequenced on a PromethION sequencer (Oxford Nanopore Technologies, Oxford, UK) for about 48–72 h.

For Illumina sequencing, poly(A) RNA was also used to construct the library using the Illumina TruSeq stranded RNA kit (Catalog No. 20020594, Illumina, San Diego, CA), following the manufacturer’s recommendations. Transcriptome sequencing of the prepared libraries was performed on an Illumina NovaSeq platform with paired-end 150 bp reads (Novogene, Beijing, China).

### Base calling, filtering, and mapping

The raw reads containing continuous current traces from the ONT sequencer were stored in FAST5 format. These reads were base-called on GUPPY (version 3.2.6) software using default RNA parameters and then covered to fastq format using the seqkit tool (version 0.11.0) [64]. The raw fastq reads were filtered by NanoFilt (version 2.6.0) with parameters “-q 7 -l 50” [65]. The passed reads were firstly corrected by filtering short reads using FMLRC (version 2) [66] and then aligned with the Nipponbare reference genome (version 7.0) [43] through minimap2 (version 2.17) [67] to obtain the consensus and non-redundant sequence using Flair (version 1.4.0) [68]. StringTie (version 2.1.2) [50] was applied to combine the aligned sequences, thus producing the novel reference transcript file for the rice genome, and GffCompare [51] was utilized to analyze the novel transcripts derived from ONT DRS. The read coverage along the chromosome was displayed using integrative genomics viewer (IGV) tools [69].

### Calculation of DEGs and DEIs from DRS data

The consensus reads obtained by DRS were mapped to novel reference transcripts using minimap2 (version 2.17) with parameters “-a -k14 -uf -x splice --secondary = no” [67], and the resulting files were submitted to salmon tools to quantify expression at the gene and transcript levels [52]. The adjusted *P* values were calculated using the Benjamini–Hochberg method [70] to control the FDR (false discovery rate). The expression levels of the genes and transcripts were expressed as TPM. DEGs and DEIs were defined as |log_2_ fold change| > 1 and adjusted *P* < 0.05.

### Expression profiling of Illumina sequencing datasets

All 12 Illumina sequencing datasets were assessed for quality using FastQC (version 0.11.3) and filtered using Trimmomatic (version 0.38) [71] to obtain clean data. The clean reads were aligned to the Nipponbare reference genome (version 7.0) [43] using Hisat2 [72] with default parameters. FeatureCount (version 1.6.4) [73] in the Rsubread package was used to obtain the read count and TPM value of each expressed gene. A differential expression analysis between pairs of samples was performed using the DESeq2 R package [74].

### Poly(A) tail length estimation

The poly(A) tail length of each read was estimated from the raw signal using Nanopolish (version 0.12.5) with parameter polya [37]. Only the poly(A) length that passed quality control according to nanopolish was further considered for estimation. The median of each transcript from all reads represented the poly(A) tail length.

### RNA base modification detection and analysis

The pass reads of FAST5 files were converted to single-read format using ont_fast5_api (version 3.1.6) with parameter “--recursive”, which were then aligned through default resquiggle in Tombo (version 1.5) [53] with a transcript reference, in which the pipeline of mappy [67] was applied to align and allocate these reads onto specific isoforms. The modifications of m^5^C and m^6^A in these specific isoforms were further identified. Models of ‘m^5^C’ and ‘*de novo*’ in Tombo were used separately to detect possible modifications in each read. The scores on each site indicated the fraction and coverage of a possible modification on a given site. The sites with fraction > 0.7 and coverage > 10 were selected for further analysis. The nine bases surrounding the modified C were used to analyze the conserved motif through MEME [75]. For m^6^A detection, MINES tool (cDNA_MINES.py) [76] with default parameters was implemented to detect m^6^A modification based on the *de novo* model, in which all regions containing a DRACH motif were identified and a new set of regions was generated by extending 10 bp on both sides of the “A” within the DRACH motifs. These regions with coverage > 5 were filtered and subjected to further analysis. The MetaPlotR package [77] was applied to draw metagene plots of the modification coverage along gene body and UTRs.

### Identification of putative m^6^A methyltransferase in the rice genome

The protein sequences of six m^6^A methyltransferases in *Arabidopsis* (AtMTA, AtMTB, AtMTC, AtFIP37, AtVIR, and AtHAKAI) and five m^6^A methyltransferases in humans (METTL3, METTL14, WTAP, KIAA1429, and HAKAI) [54] were downloaded from the TAIR database (https://www.arabidopsis.org/) and the National Center for Biotechnology Information (https://www.ncbi.nlm.nih.gov/), respectively. These proteins were used as queries to blast against the rice protein database through BLASTP, and the proteins with E value < 1E−5 and identity > 40% were screened as candidates. As a result, eight putative m^6^A methyltransferases were identified: OsMETTL14-1 (LOC_Os01g16180, homologous to METTL14), OsMETTL14-2 (LOC_Os03g05420, homologous to METTL14), OsMETTL14-3 (LOC_Os10g31030, homologous to METTL14), OsMETTL3 (LOC_Os02g45110, homologous to METTL3), OsMTC (LOC_Os03g10224, homologous to AtMTC), OsFIP37 (LOC_Os06g27970, homologous to AtFIP37), OsVIR (LOC_Os03g35340, homologous to AtVIR), and OsHAKAI (LOC_Os10g35190, homologous to AtHAKAI).

### Functional enrichment analysis

GO enrichment analyses of m^6^A- and m^5^C-methylated genes were conducted using the agriGO bioinformatics database with hypergeometric test and FDR adjustment [78]. Terms with FDR < 0.05 were considered significantly enriched.

### RNA base-modified genes and QTL analysis

The data, including physical positions of 8216 rice QTLs, were downloaded from Gramene (https://www.gramene.org), and only QTL intervals of < 2 Mb were selected for further analysis, resulting in 3729 QTLs. The QTL density along the chromosome was calculated in 200-kb windows. The site density of m^6^A and m^5^C modifications was also counted in 200-kb windows. The numbers of sites and corresponding genes in each QTL were analyzed. The distribution of QTLs and modified sites along the chromosome was drawn using R package “RIdeogram” [79].

### Amplification of novel-identified transcripts

The sequences of the novel-identified transcripts were subjected to designed primers (Table S3) flanking the overall length for PCR. For novel transcripts that were not from the annotated genes in the rice genome, primers were simultaneously used to amplify cDNA and genomic DNA. For novel transcripts that were from the annotated genes in the rice genome, primers of novel and annotated transcripts were simultaneously used to amplify cDNA. The genomic DNA was extracted from seedling leaves of Nipponbare using modified CTAB methods [80], and cDNA was reverse-transcribed from purified mRNA using HiScript II Q RT SuperMix for qPCR (add gDNA wiper) (Catalog No. R233, Vazyme, Nanjing, China). The PCR products were shifted to 0.8% agarose gel. The target bands were recycled using the gel extraction kit (Catalog No. D2500, Omega, Norcross, GA), and the resulting products were inserted into the T-vector according to the TA/Blunt-Zero cloning kit (Catalog No. C601, Vazyme). The clones were sequenced using M13 primer and then further aligned to the reference sequence using CLC sequence viewer (CLC bio LLC, Cambridge, MA).

## Data availability

The raw FAST5 data have been deposited in the Genome Sequence Archive [81] at the National Genomics Data Center, Beijing Institute of Genomics, Chinese Academy of Sciences / China National Center for Bioinformation (GSA: CRA007279), which are publicly accessible at https://ngdc.cncb.ac.cn/gsa. The long read data of each sample have been deposited in the Sequence Read Archive at the National Center for Biotechnology Information (SRA: PRJNA752930).

## Competing interests

The authors have declared no competing interests.

## CRediT authorship contribution statement

**Feng Yu:** Conceptualization, Methodology, Software, Writing – original draft. **Huanhuan Qi:** Visualization, Software, Data curation. **Li Gao:** Visualization, Investigation. **Sen Luo:** Investigation. **Rebecca Njeri Damaris:** Writing – review & editing. **Yinggen Ke:** Investigation, Writing – original draft. **Wenhua Wu:** Resources, Supervision. **Pingfang Yang:** Conceptualization, Project administration, Funding acquisition, Writing – review & editing. All authors have read and approved the final manuscript.

## Supplementary material

Supplementary Figure S1 **The pictures of six tissues that were subjected to direct RNA sequencing** Leaves, stems, and roots from the two-week-old seedlings, pistil and stamen from the booting stage, and embryo from the mature dry seeds were collected. Red rectangular box and arrow indicate the sampled tissues.

Supplementary Figure S2 **The reads coverage of newly identified isoforms in Figure 2 showing by IGV software** The reads coverage of *LOC_Os01g64090* in root, *LOC_Os03g48626* in stem, *LOC_Os02g32814* in stem, *LOC_Os02g03440* in pistil, and *LOC_Os12g38051* in root were displayed. R1 and R2 represent two independent replicates. Red color indicates novel isoforms. IGV, integrative genomics viewer.

Supplementary Figure S3 **The negative controls of amplified products in Figure 2** RNA indicated that the no reversely transcribed RNA was treated as a template, cDNA was the template reversely transcribed from RNA, and DNA was the genome DNA of Nipponbare. All of the primers except actin were from Figure 2, and the primer of actin was designed from rice gene *LOC_Os03g50885.1.* S, stem; P, pistil; R, root*.*

Supplementary Figure S4 **The upset plot displayed the number of expressed genes in six tissues through direct RNA sequencing** The number of expressed genes in root, stem, stamen, pistil, leaf, and embryo were shown through upset plot.

Supplementary Figure S5 **The upset plot displayed the number of expressed isoforms in six tissues through direct RNA sequencing** The number of expressed isoforms in root, stem, stamen, pistil, leaf, and embryo were shown through upset plot.

Supplementary Figure S6 **Veen diagram showing the overlapped number of DEIs and DEGs in each comparison** The number of differentially expressed isoforms and genes were compared among six tissues of root, stem, stamen, pistil, leaf, and embryo. DEIs, differentially expressed isoforms; DEGs, differentially expressed genes.

Supplementary Figure S7 **Comparison of the differentially expressed genes identified by DRS and NGS** The differentially expressed genes that detected by DRS and NGS in comparison of embryo with leaf, root, embryo, stamen, and stem, leaf with stamen, and root with stamen were displayed. DRS, direct RNA sequencing; NGS, next-generation sequencing (Illumina).

Supplementary Figure S8 **Comparison of the repeatability of m6A identification in six tissues A.** Comparison of the m6A sites between two replications. **B.** Comparison of the genes modified by m6A between two replications. **C.** Correlation analysis for the fraction of the overlapped sites in two replications. Rep 1, replicate 1; Rep 2, replicate 2.

Supplementary Figure S9 **Comparison of the repeatability of m^5^C identification in six tissues A.** Comparison of the m^5^C sites between two replications. **B.** Comparison of the genes modified by m^5^C between two replications. **C.** Correlation analysis for the fraction of the overlapped sites in two replications. Rep1, replicate 1; Rep2, replicate 2.

Supplementary Figure S10 **Comparison of the m^6^A modification identified by DRS with previous identification through MeRIP in the root A.** Comparison of the m^6^A-modified sites in DRS data with genomic regions identified by MeRIP. **B.** Comparison of the m^6^A-modified genes in DRS data with modified genes detected by MeRIP. The sites and genes that overlapped in two replications were used; Rep1, replicate 1; Rep2, replicate 2; MeRIP, methylated RNA immunoprecipitation sequencing.

Supplementary Figure S11 **Comparing the fraction of modified sites in all transcripts with these falling into > 5 categories in Figure 4B** The fraction of m^6^A-modified sites in each transcript was calculated, and the maximum fraction in each transcript was counted.

Supplementary Figure S12 **The percentage of m6A-modified motifs in the six tissues** The percentage of four conserved motifs surrounding the modified base A in root, stem, stamen, pistil, leaf, and embryo was calculated, respectively.

Supplementary Figure S13 **Comparison of the fraction of modified sites in all transcripts with those falling into > 15 categories in Figure 5B** The fraction of m^5^C-modified sites in each transcript was calculated, and the maximum fraction in each transcript was counted.

Supplementary Figure S14 **The significantly enriched motif around the modified base C through MEME analysis** Four conserved motifs surrounding the modified base C were identified and the corresponding site number wal calculated.

Supplementary Figure S15 **The proportion of modified and non-modified transcripts of modified genes** The ratio of m^6^A and m^5^C modified transcripts in modified genes in tissue of root, stem, stamen, pistil, leaf, and embryo was shown, respectively.

Supplementary Figure S16 **The expression level of transcripts with different sites of m6A modification** The m^6^A modified transcripts were divided into three categories based on modified site number, and the expression level of each category in root, stem, stamen, pistil, leaf, and embryo was calculated, respectively. TPM, transcripts per kilobase per million.

Supplementary Figure S17 **The expression level of transcripts with different sites of m^5^C modification** The m^5^C modified transcripts were divided into six categories based on modified site number, and the expression level of each category in root, stem, stamen, pistil, leaf, and embryo was calculated, respectively. TPM, transcripts per kilobase per million.

Supplementary Figure S18 **The poly(A) tail length of transcripts with the different number of m^6^A modification sites** The m^6^A modified transcripts were divided into three categories based on modified site number, and the polyA tail length of each category in root, stem, stamen, pistil, leaf, and embryo was calculated, respectively.

Supplementary Figure S19 **The poly(A) tail length of transcripts with the different number of m^5^C modification sites** The m^5^C modified transcripts were divided into six categories based on modified site number, and the polyA tail length of each category in root, stem, stamen, pistil, leaf, and embryo was calculated, respectively.

Supplementary Figure S20 **The distribution of modification sites in transcript *LOC_Os06g40360.1* A.** The m^6^A and m^5^C modified sites in each tissue distributed within *LOC_Os06g40360.1*. **B.** Ionic current signal of each nucleoside in transcript *LOC_Os06g40360.1*. The position indicated the transcript length from 2645 to 2669, m indicated the methylated A or C, red color indicated the nucleoside signal, and black color indicated the model.

Supplementary Figure S21 **The GO analysis of commonly modified transcripts in all six tissues through AgriGO** The left panel showed GO terms of 4420 transcripts that were commonly modified by m^6^A, and the right panel showed GO terms of 2983 transcripts that were commonly modified by m^5^C. The significant GO terms were selected by FDR < 0.05. GO, gene ontology; FDR, false discovery rate.

Supplementary Figure S22 **The GO analysis of commonly modified transcripts by m^6^A and m^5^C in six tissues in Figure 5G through AgriGO** The transcripts that commonly modified by m^6^A and m^5^C in root, stem, stamen, pistil, leaf, and embryo were subjected to enrich the GO terms, respectively. The significant GO terms were selected by FDR < 1E–05.

Supplementary Figure S23 **The GO analysis of specifically modified transcripts by m^6^A and m^5^C in each tissue in Figure 5G through AgriGO** The transcripts that specifically modified by m^6^A and m^5^C in root, stem, stamen, pistil, leaf, and embryo were subjected to enrich the GO terms, respectively. The significant GO terms were selected by FDR < 1E–04.

Supplementary Table S1 **Statistical information of Nanopore native RNA clean reads in all sequenced samples**

Supplementary Table S2 **The information of novel identified transcripts through stringTie**

Supplementary Table S3 **The sequence information for amplifying the novel transcripts identified by direct RNA sequencing**

Supplementary Table S4 **The sequence of newly identified transcripts that were sequenced by Sanger technique**

Supplementary Table S5 **The detailed information of detected m^6^A sites in six tissues**

Supplementary Table S6 **The detailed information of detected m^5^C sites in six tissues**

Supplementary Table S7 **The correlation between expression level or poly(A) tail length of transcripts and the location of modified sites**
