# Supplementary figures and images for: Identifying RNA Modifications by Direct RNA Sequencing Reveals Complexity of Epitranscriptomic Dynamics in Rice
Source: Genomics Proteomics Bioinformatics. 2023 Feb 11;21(4):788–804. doi: 10.1016/j.gpb.2023.02.002 (PMC10787127; doi:10.1016/j.gpb.2023.02.002)

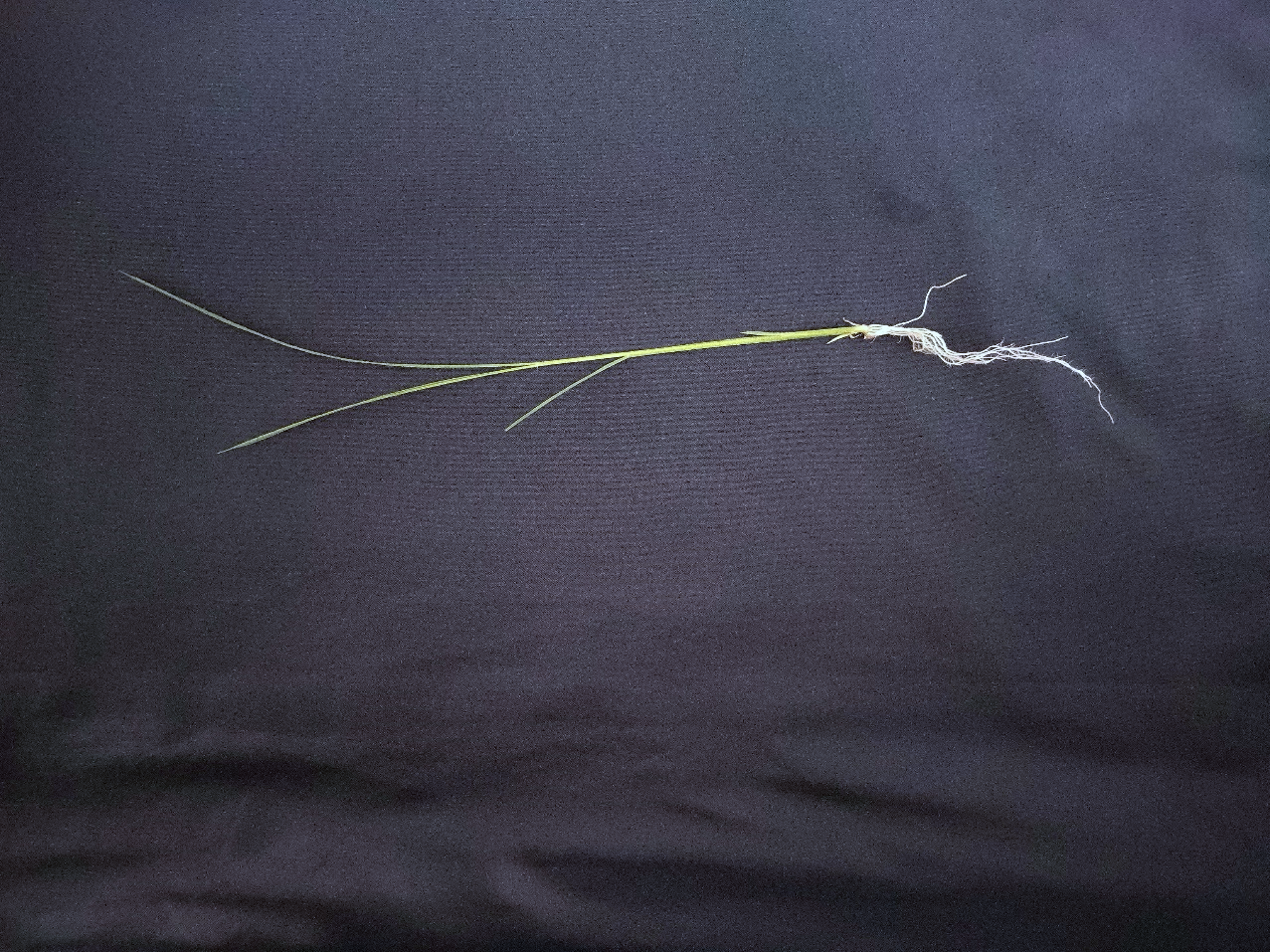

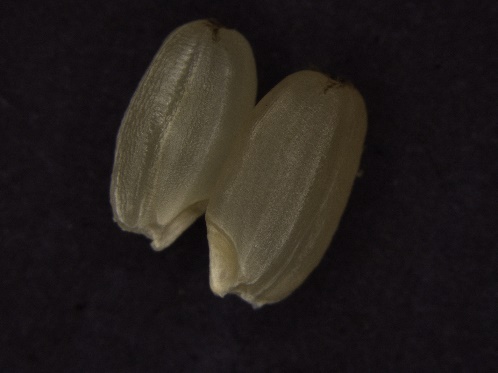

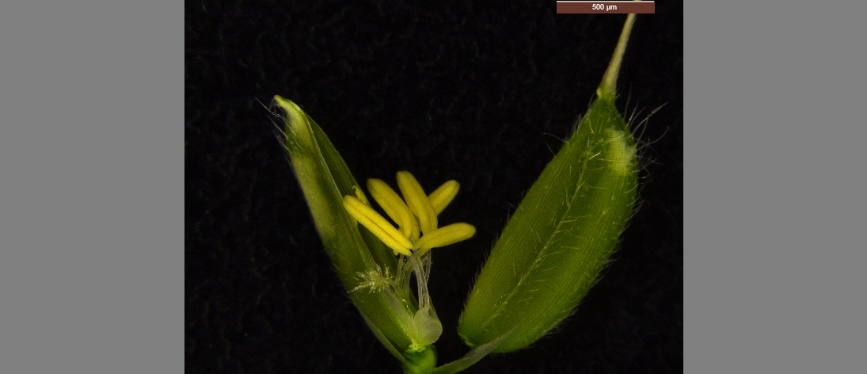


Leaf

Stem

Root

Stamen

Pistil

Embryo

Supplement: Supplementary Figure S1 — The pictures of six tissues that were subjected to direct RNA sequencing Leaves, stems, and roots from the two-week-old seedlings, pistil and stamen from the booting stage, and embryo from the mature dry seeds were collected. Red rectangular box and arrow indicate the sampled tissues. [file mmc1.docx]

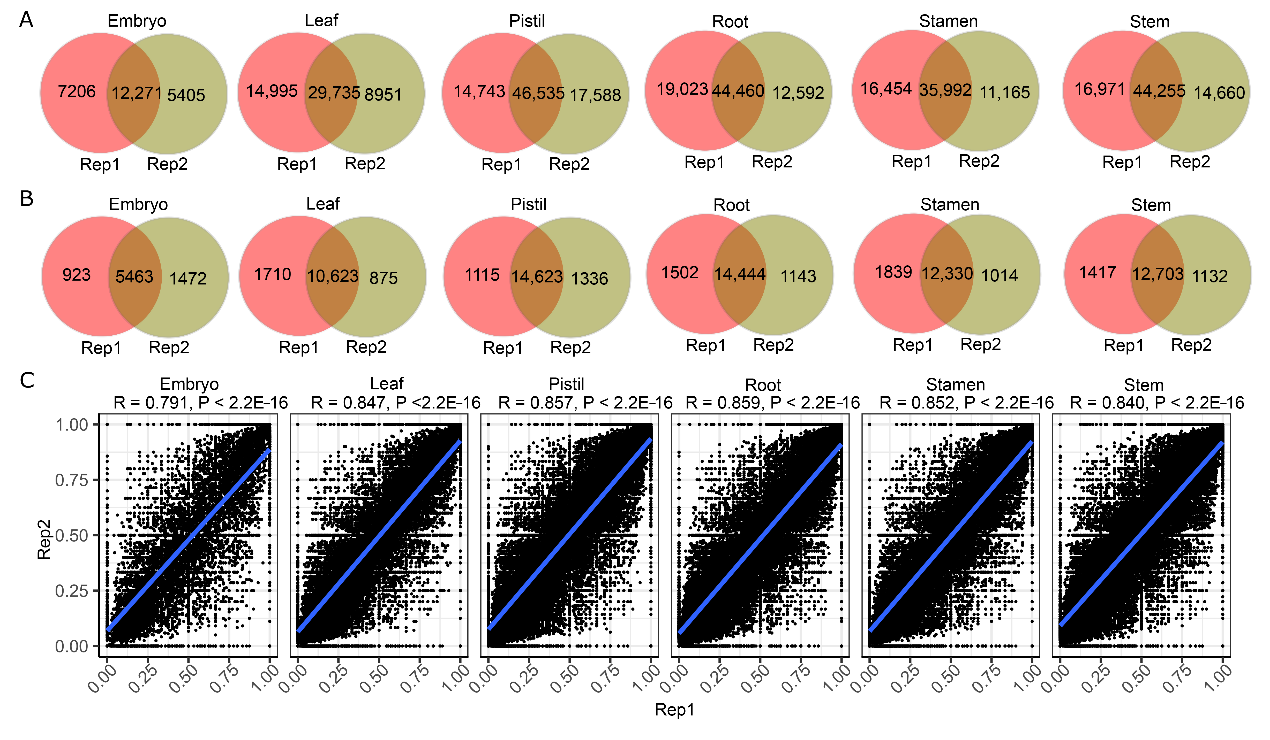

Supplement: Supplementary Figure S8 — Comparison of the repeatability of m6A identification in six tissues A. Comparison of the m6A sites between two replications. B. Comparison of the genes modified by m6A between two replications. C. Correlation analysis for the fraction of the overlapped sites in two replications. Rep 1, replicate 1; Rep 2, replicate 2. [file mmc8.docx]

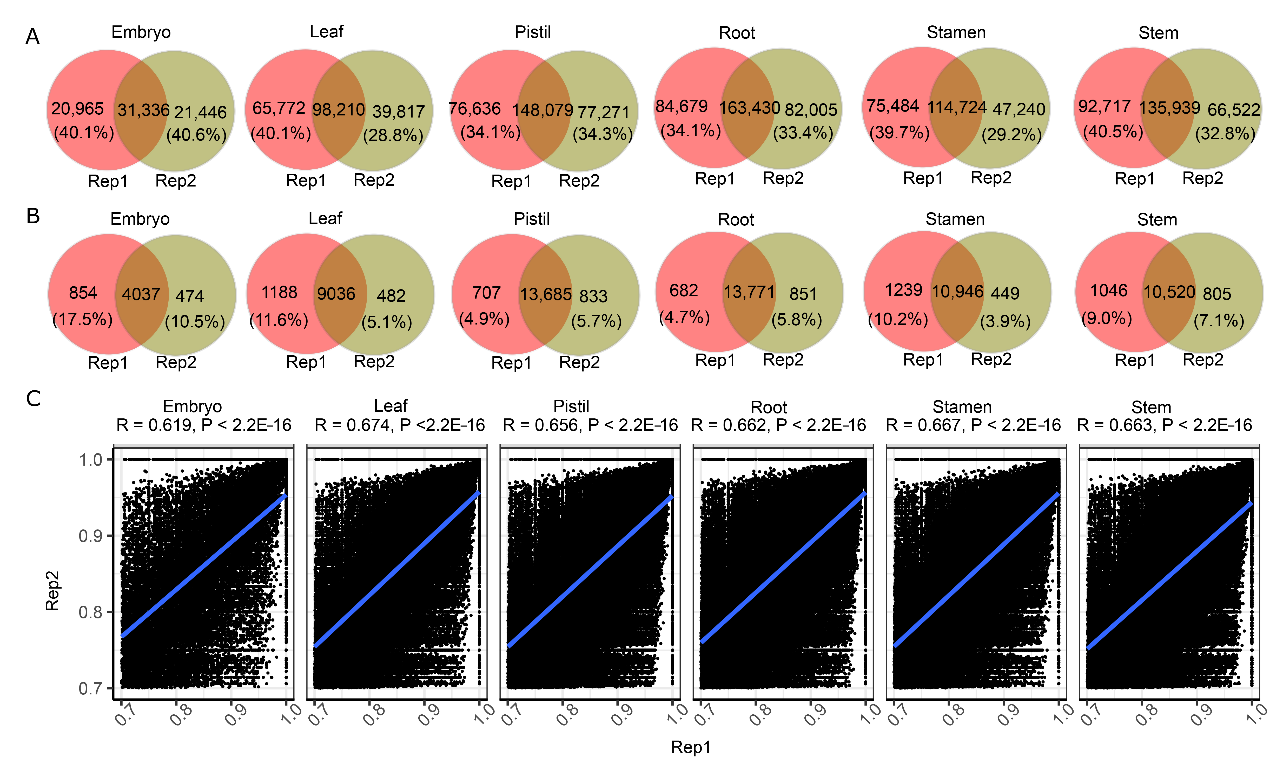

Supplement: Supplementary Figure S9 — Comparison of the repeatability of m5C identification in six tissues A. Comparison of the m5C sites between two replications. B. Comparison of the genes modified by m5C between two replications. C. Correlation analysis for the fraction of the overlapped sites in two replications. Rep1, replicate 1; Rep2, replicate 2. [file mmc9.docx]

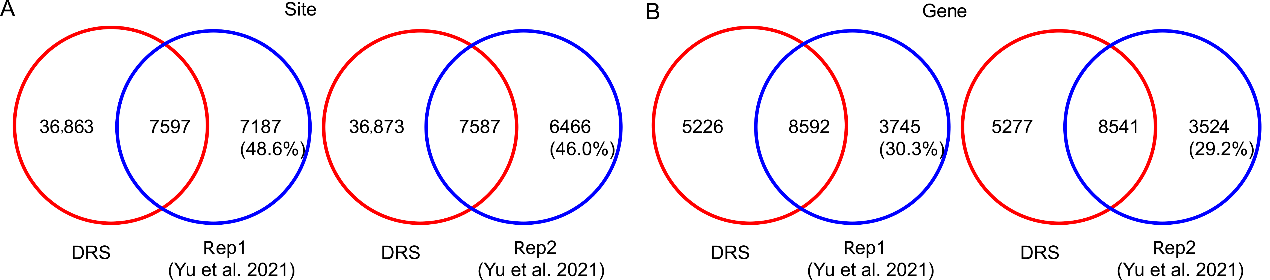

Supplement: Supplementary Figure S10 — Comparison of the m6A modification identified by DRS with previous identification through MeRIP in the root A. Comparison of the m6A-modified sites in DRS data with genomic regions identified by MeRIP. B. Comparison of the m6A-modified genes in DRS data with modified genes detected by MeRIP. The sites and genes that overlapped in two replications were used; Rep1, replicate 1; Rep2, replicate 2; MeRIP, methylated RNA immunoprecipitation sequencing. [file mmc10.docx]

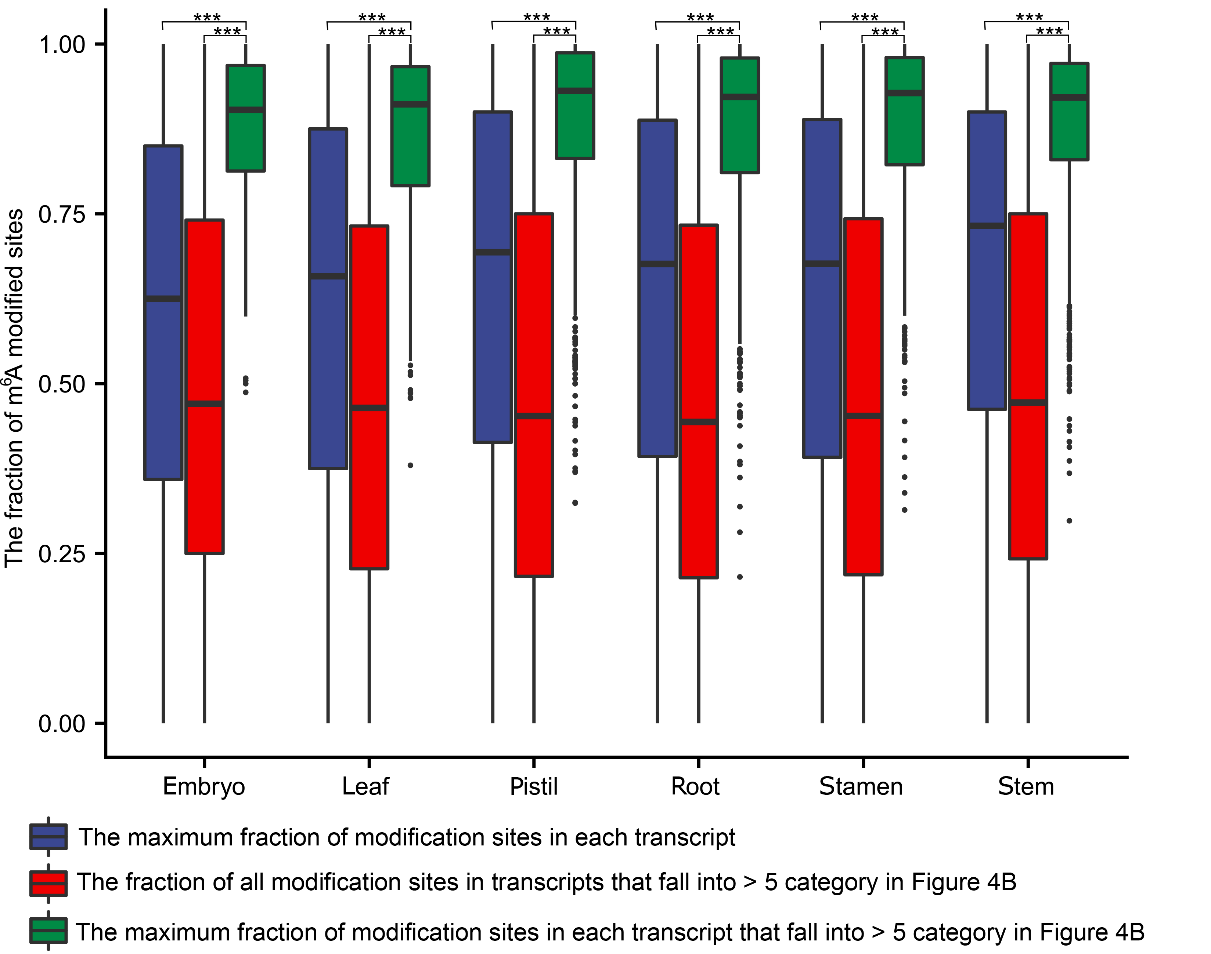

Supplement: Supplementary Figure S11 — Comparing the fraction of modified sites in all transcripts with these falling into > 5 categories in Figure 4B The fraction of m6A-modified sites in each transcript was calculated, and the maximum fraction in each transcript was counted. [file mmc11.docx]

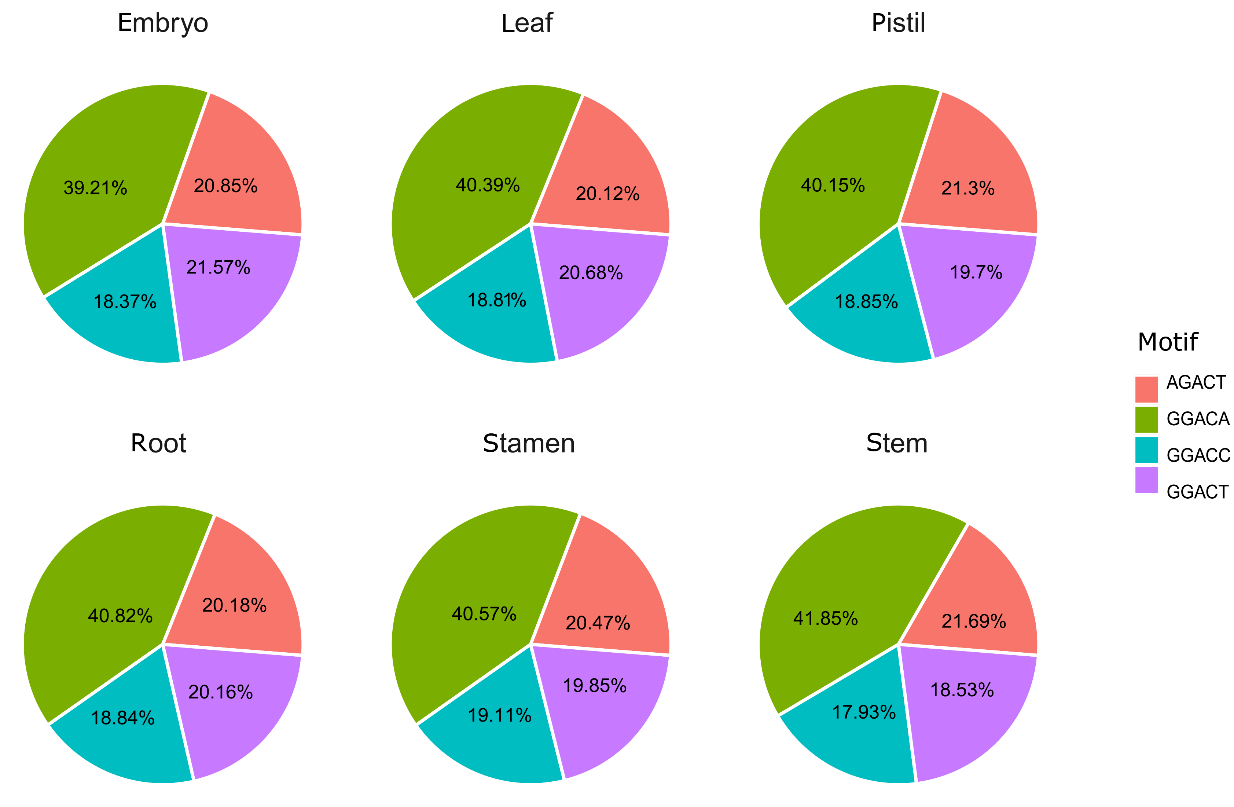

Supplement: Supplementary Figure S12 — The percentage of m6A-modified motifs in the six tissues The percentage of four conserved motifs surrounding the modified base A in root, stem, stamen, pistil, leaf, and embryo was calculated, respectively. [file mmc12.docx]

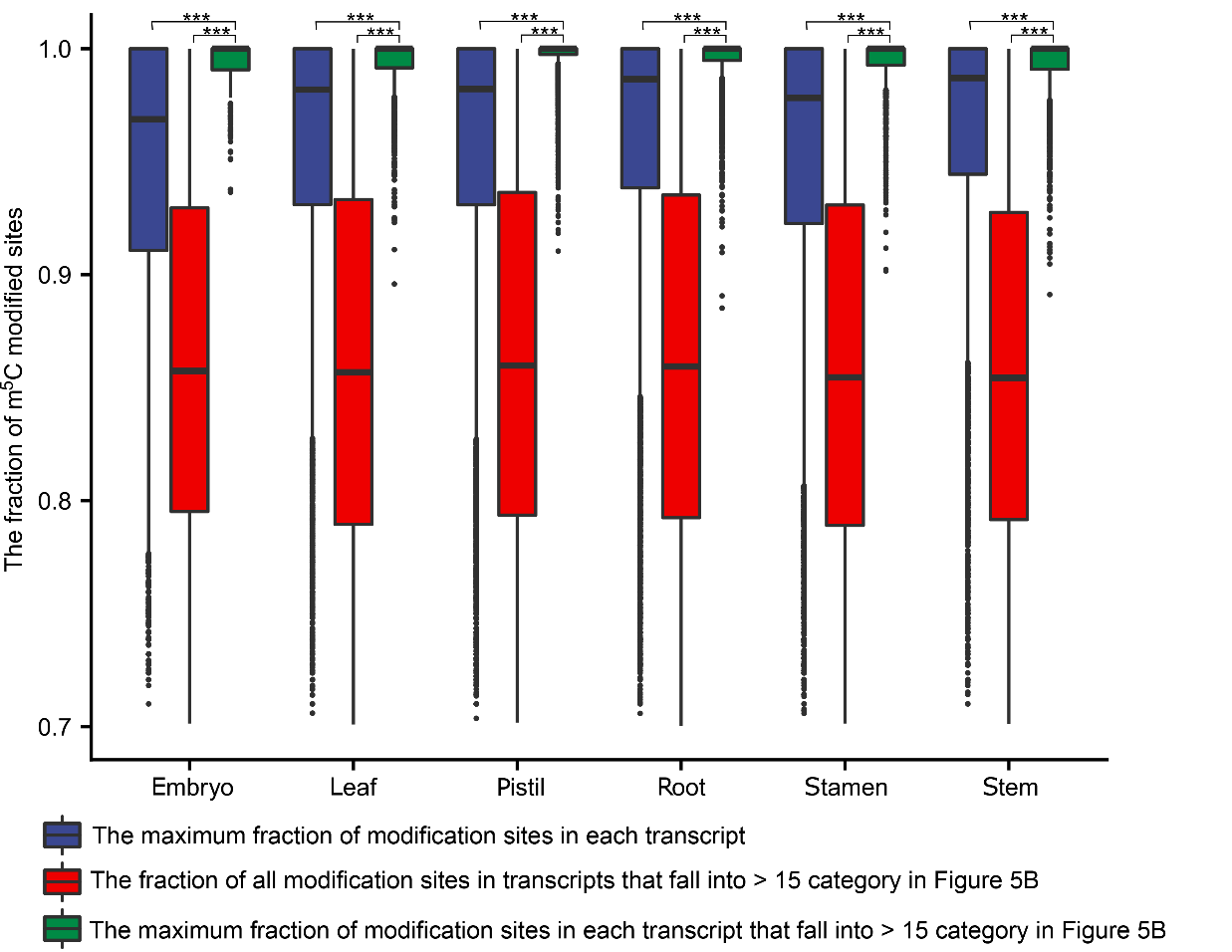

Supplement: Supplementary Figure S13 — Comparison of the fraction of modified sites in all transcripts with those falling into > 15 categories in Figure 5B The fraction of m5C-modified sites in each transcript was calculated, and the maximum fraction in each transcript was counted. [file mmc13.docx]

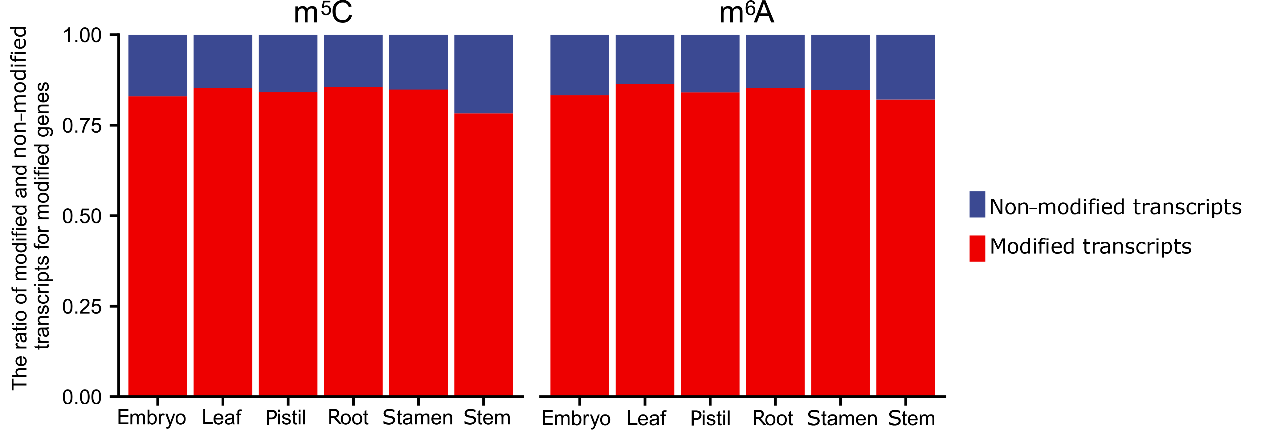

Supplement: Supplementary Figure S15 — The proportion of modified and non-modified transcripts of modified genes The ratio of m6A and m5C modified transcripts in modified genes in tissue of root, stem, stamen, pistil, leaf, and embryo was shown, respectively. [file mmc15.docx]

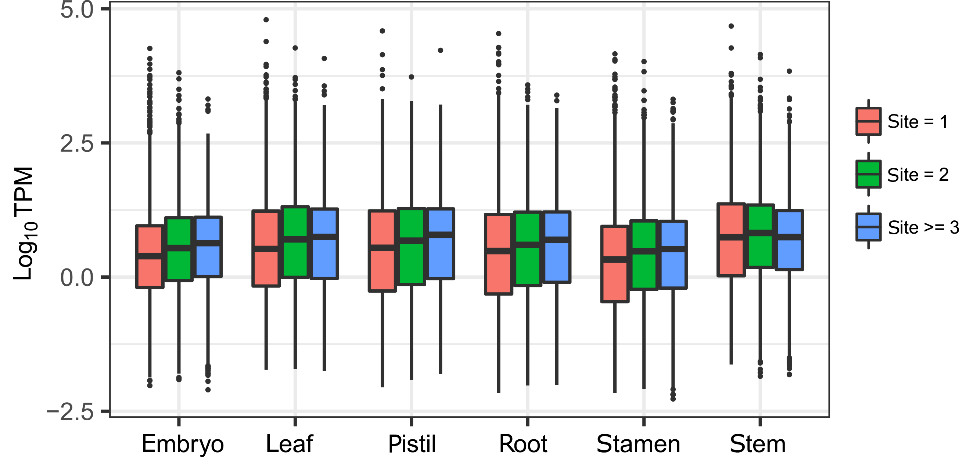

Supplement: Supplementary Figure S16 — The expression level of transcripts with different sites of m6A modification The m6A modified transcripts were divided into three categories based on modified site number, and the expression level of each category in root, stem, stamen, pistil, leaf, and embryo was calculated, respectively. TPM, transcripts per kilobase per million. [file mmc16.docx]

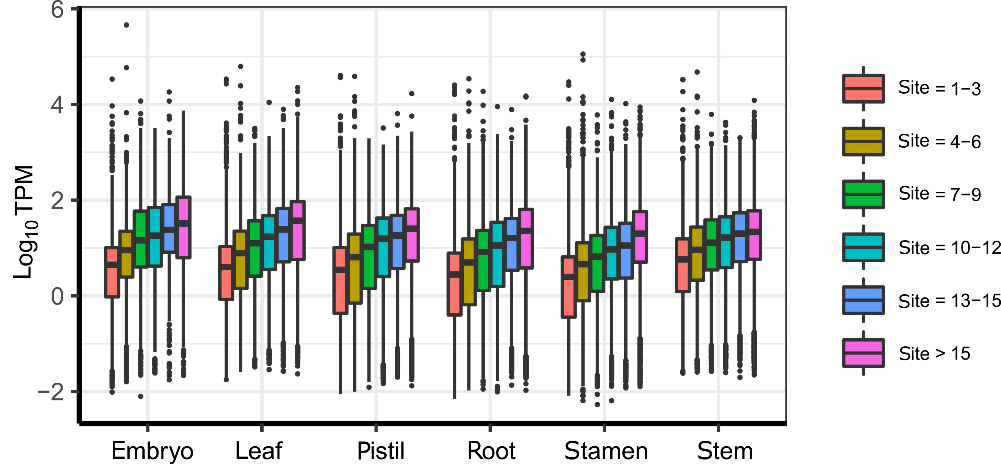

Supplement: Supplementary Figure S17 — The expression level of transcripts with different sites of m5C modification The m5C modified transcripts were divided into six categories based on modified site number, and the expression level of each category in root, stem, stamen, pistil, leaf, and embryo was calculated, respectively. TPM, transcripts per kilobase per million. [file mmc17.docx]

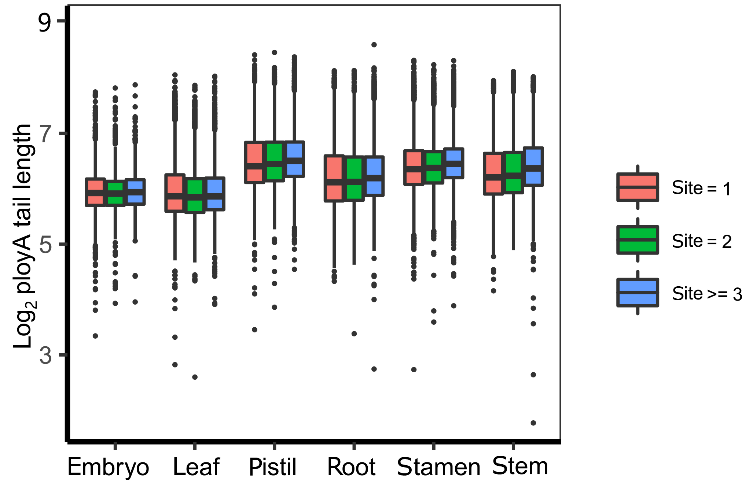

Supplement: Supplementary Figure S18 — The poly(A) tail length of transcripts with the different number of m6A modification sites The m6A modified transcripts were divided into three categories based on modified site number, and the polyA tail length of each category in root, stem, stamen, pistil, leaf, and embryo was calculated, respectively. [file mmc18.docx]

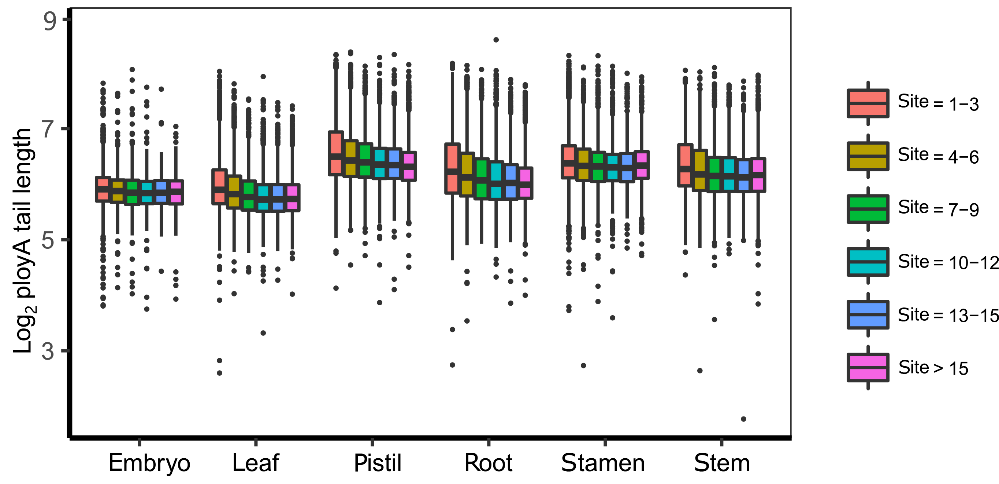

Supplement: Supplementary Figure S19 — The poly(A) tail length of transcripts with the different number of m5C modification sites The m5C modified transcripts were divided into six categories based on modified site number, and the polyA tail length of each category in root, stem, stamen, pistil, leaf, and embryo was calculated, respectively. [file mmc19.docx]

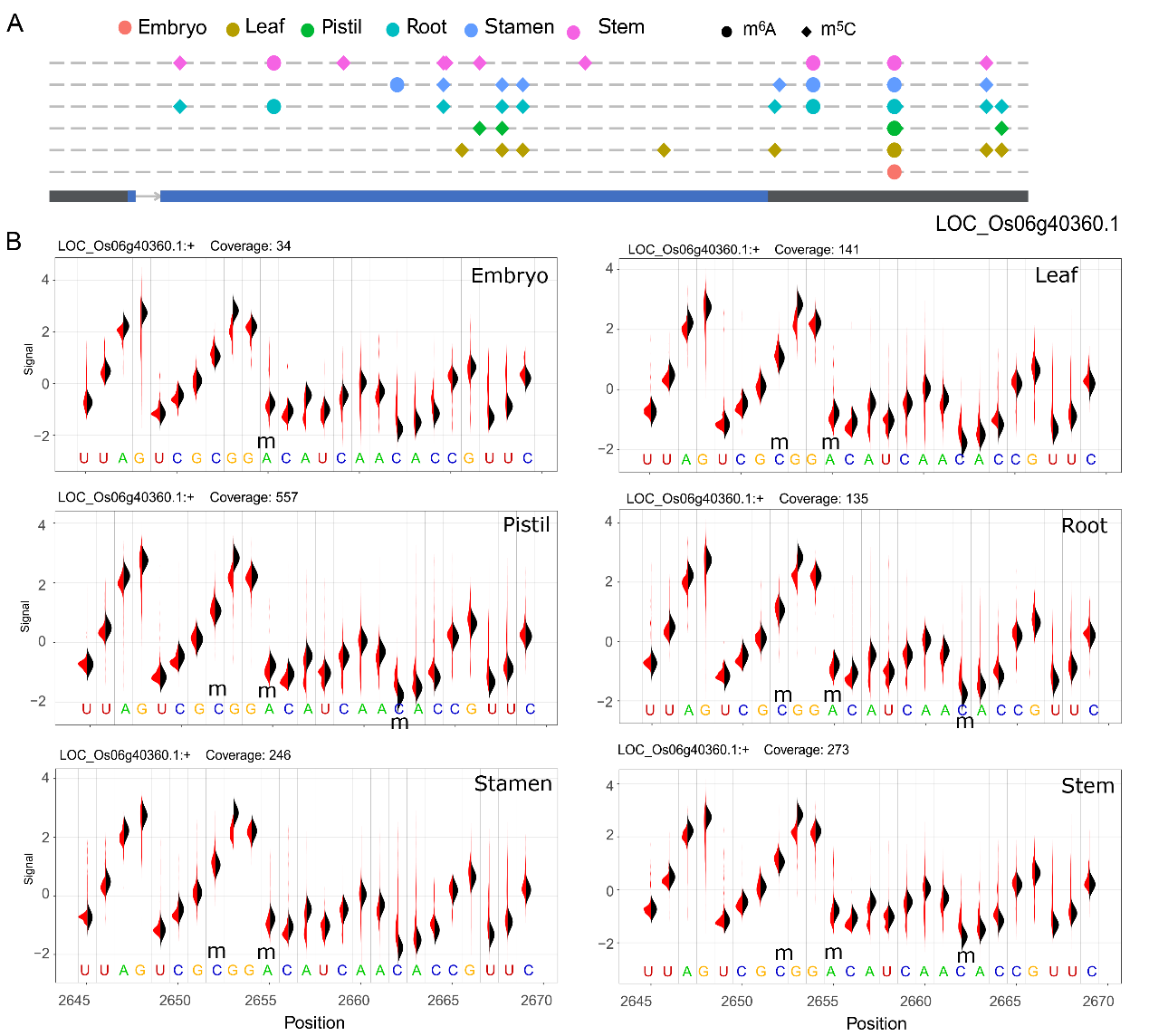

Supplement: Supplementary Figure S20 — The distribution of modification sites in transcript LOC_Os06g40360.1 A. The m6A and m5C modified sites in each tissue distributed within LOC_Os06g40360.1. B. Ionic current signal of each nucleoside in transcript LOC_Os06g40360.1. The position indicated the transcript length from 2645 to 2669, m indicated the methylated A or C, red color indicated the nucleoside signal, and black color indicated the model. [file mmc20.docx]

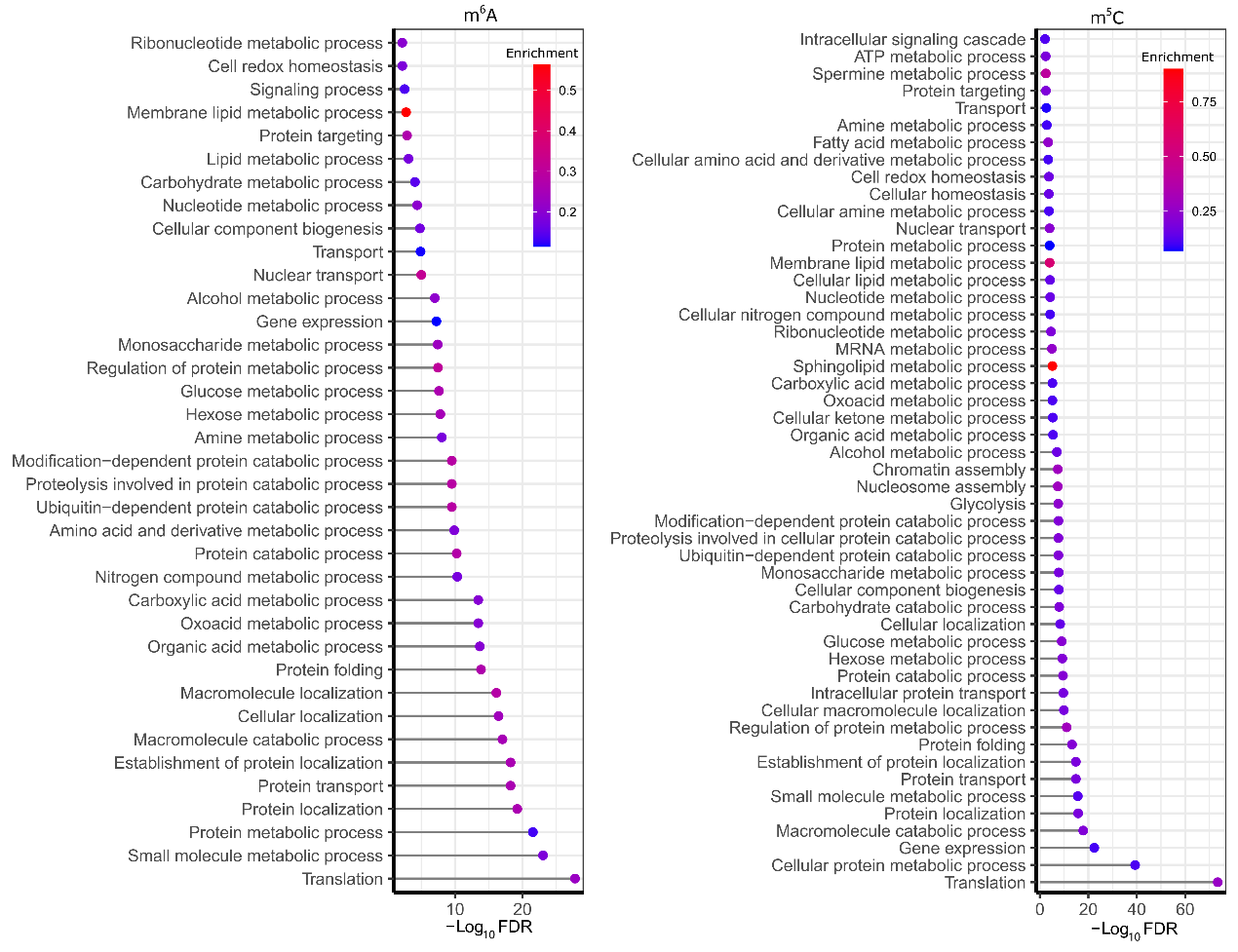

Supplement: Supplementary Figure S21 — The GO analysis of commonly modified transcripts in all six tissues through AgriGO The left panel showed GO terms of 4420 transcripts that were commonly modified by m6A, and the right panel showed GO terms of 2983 transcripts that were commonly modified by m5C. The significant GO terms were selected by FDR < 0.05. GO, gene ontology; FDR, false discovery rate. [file mmc21.docx]

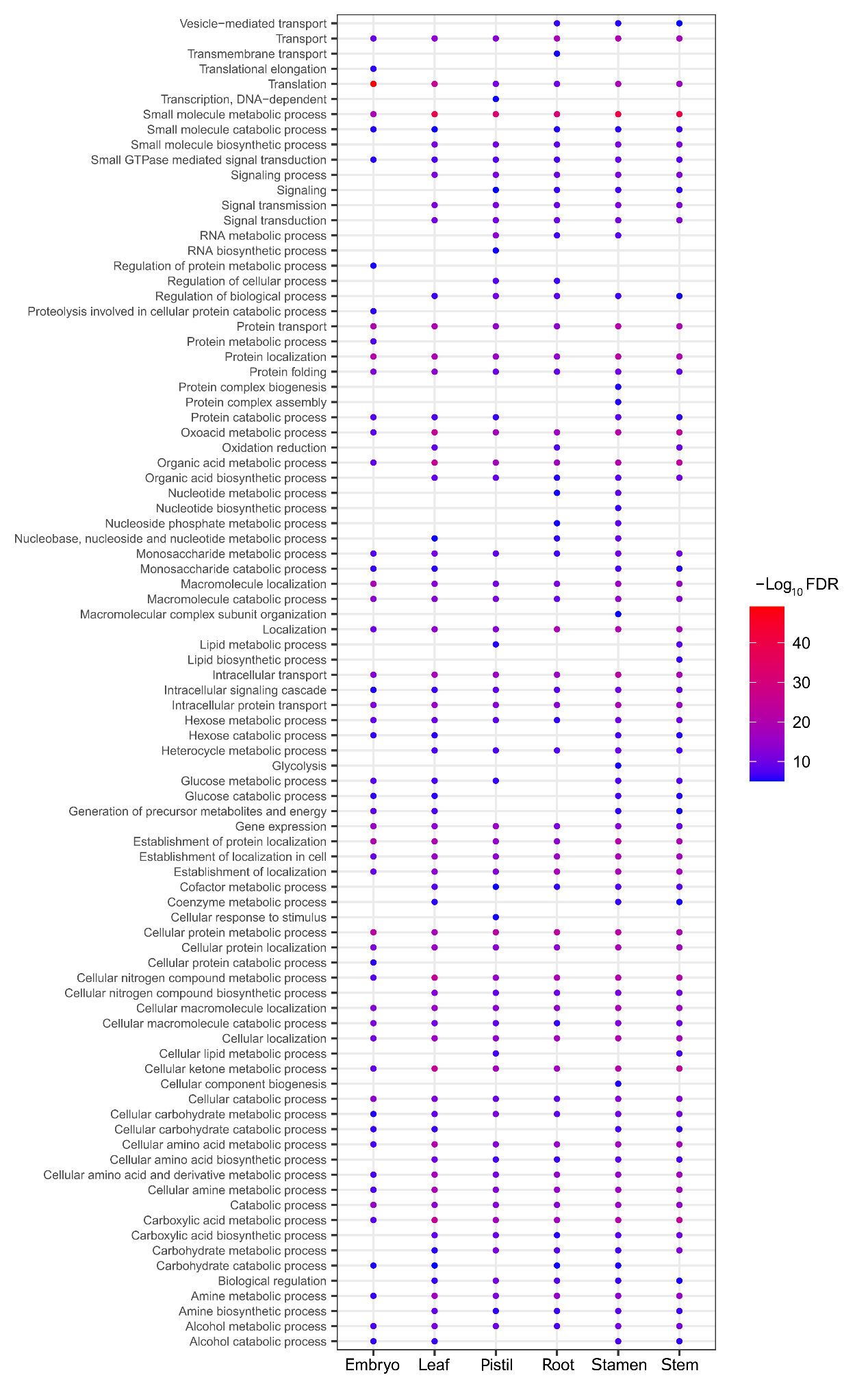

Supplement: Supplementary Figure S22 — The GO analysis of commonly modified transcripts by m6A and m5C in six tissues in Figure 5G through AgriGO The transcripts that commonly modified by m6A and m5C in root, stem, stamen, pistil, leaf, and embryo were subjected to enrich the GO terms, respectively. The significant GO terms were selected by FDR < 1E–05. [file mmc22.docx]

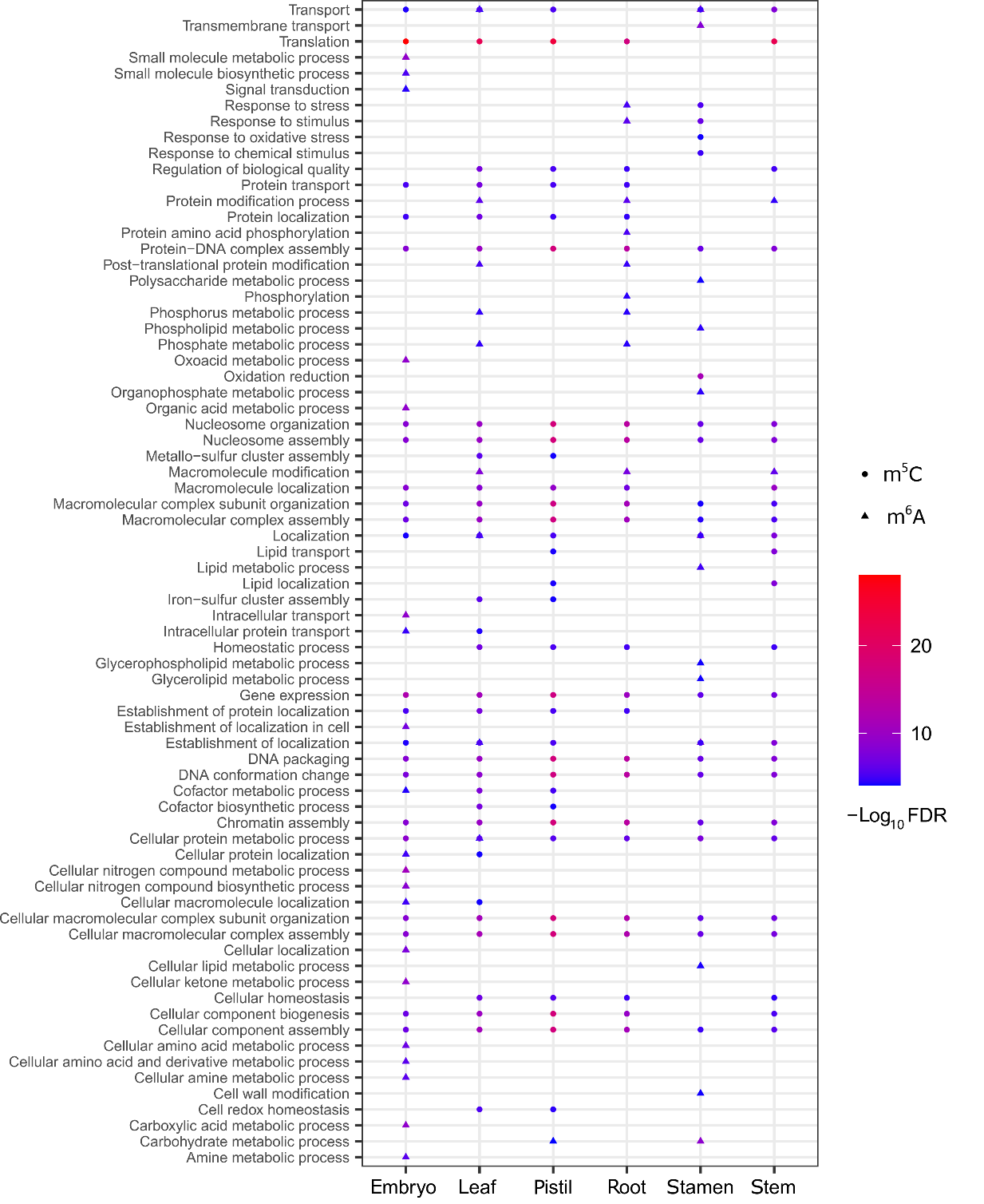

Supplement: Supplementary Figure S23 — The GO analysis of specifically modified transcripts by m6A and m5C in each tissue in Figure 5G through AgriGO The transcripts that specifically modified by m6A and m5C in root, stem, stamen, pistil, leaf, and embryo were subjected to enrich the GO terms, respectively. The significant GO terms were selected by FDR < 1E–04. [file mmc23.docx]
