# Supplementary material for: Identifying RNA Modifications by Direct RNA Sequencing Reveals Complexity of Epitranscriptomic Dynamics in Rice
Source: Genomics Proteomics Bioinformatics. 2023 Feb 11;21(4):788–804. doi: 10.1016/j.gpb.2023.02.002 (PMC10787127; doi:10.1016/j.gpb.2023.02.002)
Supplement: Supplementary file 2 [file mmc14.docx]

Number of sites

nsite = 96434

nsite = 7798

Number of sites

nsite = 17989

nsite = 2463
