# Supplementary material for: Identifying RNA Modifications by Direct RNA Sequencing Reveals Complexity of Epitranscriptomic Dynamics in Rice
Source: Genomics Proteomics Bioinformatics. 2023 Feb 11;21(4):788–804. doi: 10.1016/j.gpb.2023.02.002 (PMC10787127; doi:10.1016/j.gpb.2023.02.002)
Supplement: Supplementary file 3 [file mmc2.docx]

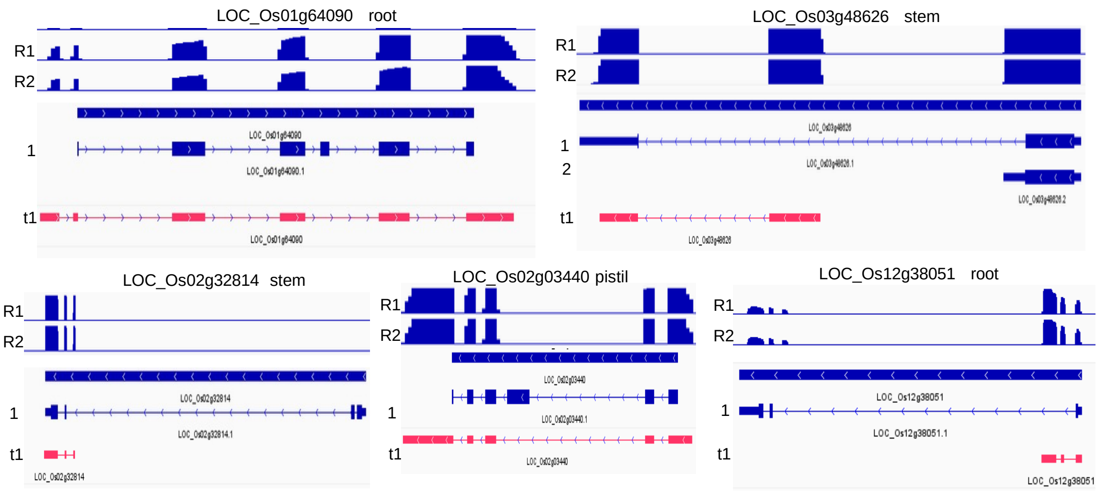
LOC_Os01g64090 root
R1
R2
1
t1
LOC_Os03g48626 stem
R1
R2
1
2
t1
LOC_Os02g32814 stem
R1
R2
1
t1
LOC_Os02g03440 pistil
R1
R2
1
t1
LOC_Os12g38051 root
R1
R2
LOC_Os12g38051
1
LOC_Os12g38051.1
t1
LOC_Os12g38051
