# Supplementary material for: Identifying RNA Modifications by Direct RNA Sequencing Reveals Complexity of Epitranscriptomic Dynamics in Rice
Source: Genomics Proteomics Bioinformatics. 2023 Feb 11;21(4):788–804. doi: 10.1016/j.gpb.2023.02.002 (PMC10787127; doi:10.1016/j.gpb.2023.02.002)
Supplement: Supplementary file 4 [file mmc3.docx]

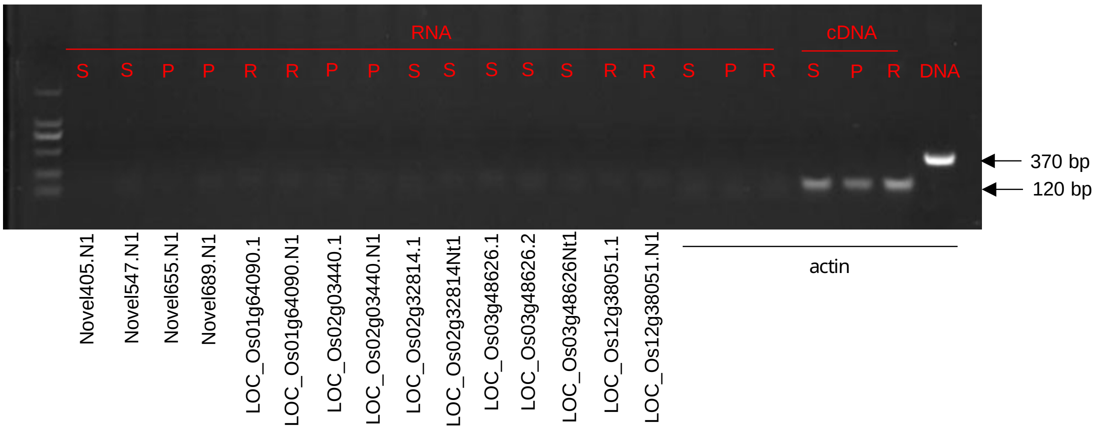
RNA
cDNA
S S P P R R P P S S S S S R R S P R
S P R
DNA
370 bp
120 bp
Novel405.N1
Novel547.N1
Novel655.N1
Novel689.N1
LOC_Os01g64090.1
LOC_Os01g64090.N1
LOC_Os02g03440.1
LOC_Os02g03440.N1
LOC_Os02g32814.1
LOC_Os02g32814Nt1
LOC_Os03g48626.1
LOC_Os03g48626.2
LOC_Os03g48626Nt1
LOC_Os12g38051.1
LOC_Os12g38051.N1
actin
