# Supplementary material for: Identifying RNA Modifications by Direct RNA Sequencing Reveals Complexity of Epitranscriptomic Dynamics in Rice
Source: Genomics Proteomics Bioinformatics. 2023 Feb 11;21(4):788–804. doi: 10.1016/j.gpb.2023.02.002 (PMC10787127; doi:10.1016/j.gpb.2023.02.002)
Supplement: Supplementary file 5 [file mmc4.docx]

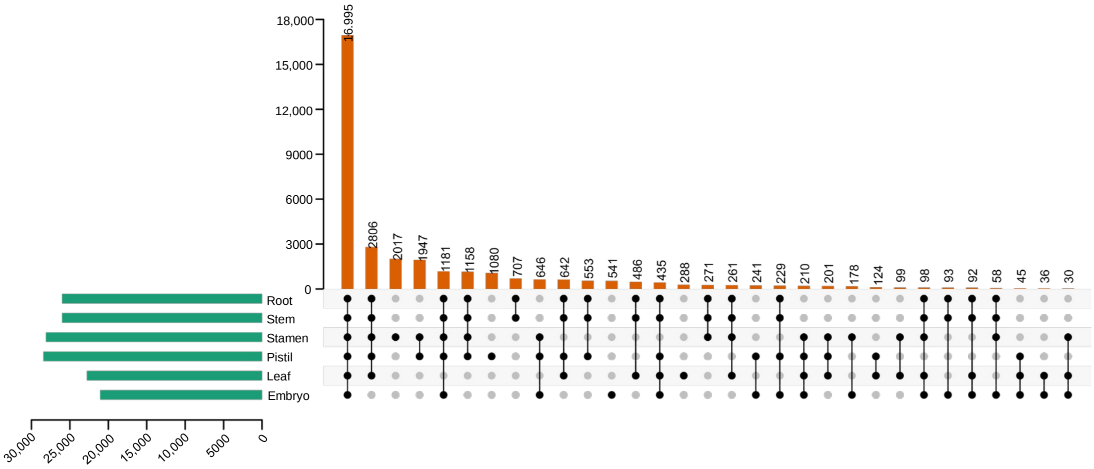

18,000
15,000
12,000
9000
6000
3000
0
16,995
2806
2017
1947
1181
1158
1080
707
646
642
553
541
486
435
288
271
261
241
229
210
201
178
124
99
98
93
92
58
45
36
30
Root
Stem
Stamen
Pistil
Leaf
Embryo
30,000
25,000
20,000
15,000
10,000
5000
0
