# Supplementary material for: Identifying RNA Modifications by Direct RNA Sequencing Reveals Complexity of Epitranscriptomic Dynamics in Rice
Source: Genomics Proteomics Bioinformatics. 2023 Feb 11;21(4):788–804. doi: 10.1016/j.gpb.2023.02.002 (PMC10787127; doi:10.1016/j.gpb.2023.02.002)
Supplement: Supplementary file 6 [file mmc5.docx]

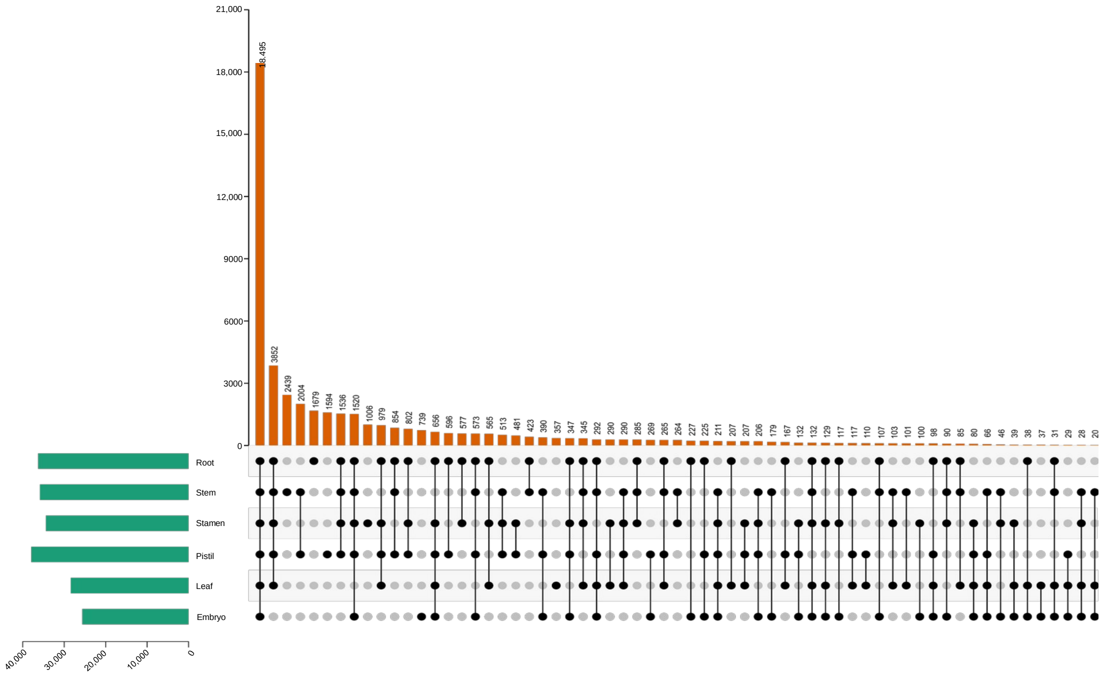
21,000
18,000
15,000
12,000
9000
6000
3000
0
18,495
3852
2439
2004
1679
1594
1536
1520
1006
979
854
802
739
656
596
577
573
565
513
481
423
390
357
347
345
292
290
290
285
269
265
264
227
225
211
207
207
206
179
167
132
132
129
117
117
110
107
103
101
100
98
90
85
80
66
46
39
38
37
31
29
28
20
Root
Stem
Stamen
Pistil
Leaf
Embryo
40,000
30,000
20,000
10,000
0
