# Supplementary material for: Identifying RNA Modifications by Direct RNA Sequencing Reveals Complexity of Epitranscriptomic Dynamics in Rice
Source: Genomics Proteomics Bioinformatics. 2023 Feb 11;21(4):788–804. doi: 10.1016/j.gpb.2023.02.002 (PMC10787127; doi:10.1016/j.gpb.2023.02.002)
Supplement: Supplementary file 7 [file mmc6.docx]

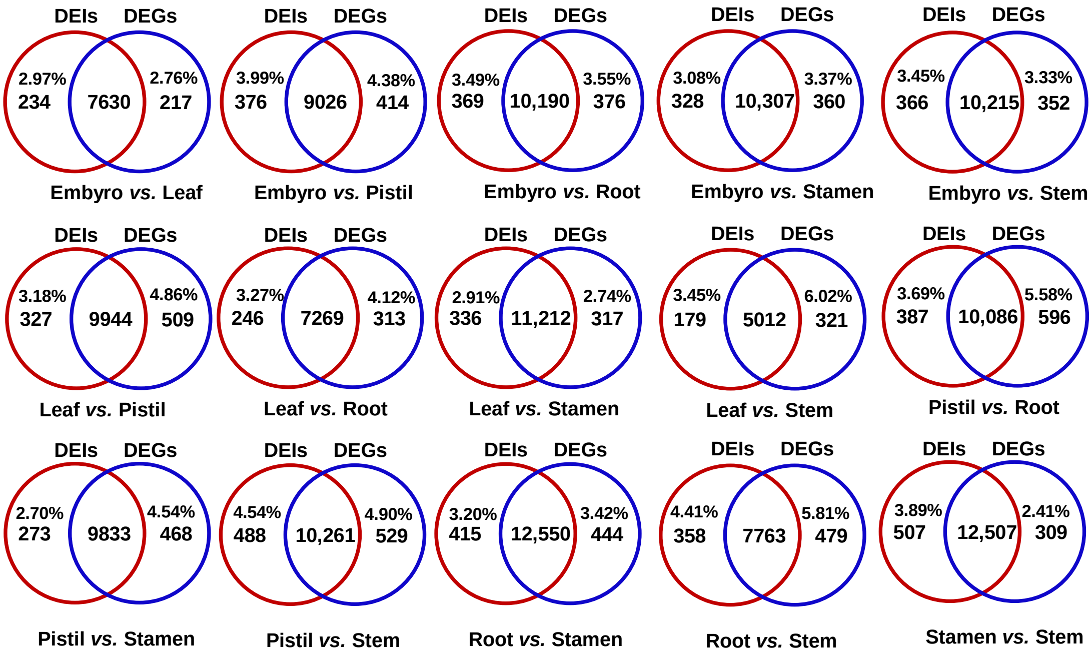
DEIs DEGs
2.97% 234 7630 2.76% 217
Embyro vs. Leaf
3.99% 376 9026 4.38% 414
Embyro vs. Pistil
3.49% 369 10,190 3.55% 376
Embyro vs. Root
3.08% 328 10,307 3.37% 360
Embyro vs. Stamen
3.45% 366 10,215 3.33% 352
Embyro vs. Stem
3.18% 327 9944 4.86% 509
Leaf vs. Pistil
3.27% 246 7269 4.12% 313
Leaf vs. Root
2.91% 336 11,212 2.74% 317
Leaf vs. Stamen
3.45% 179 5012 6.02% 321
Leaf vs. Stem
3.69% 387 10,086 5.58% 596
Pistil vs. Root
2.70% 273 9833 4.54% 468
Pistil vs. Stamen
4.54% 488 10,261 4.90% 529
Pistil vs. Stem
3.20% 415 12,550 3.42% 444
Root vs. Stamen
4.41% 358 7763 5.81% 479
Root vs. Stem
3.89% 507 12,507 2.41% 309
Stamen vs. Stem
