# Supplementary material for: Identifying RNA Modifications by Direct RNA Sequencing Reveals Complexity of Epitranscriptomic Dynamics in Rice
Source: Genomics Proteomics Bioinformatics. 2023 Feb 11;21(4):788–804. doi: 10.1016/j.gpb.2023.02.002 (PMC10787127; doi:10.1016/j.gpb.2023.02.002)
Supplement: Supplementary file 8 [file mmc7.docx]

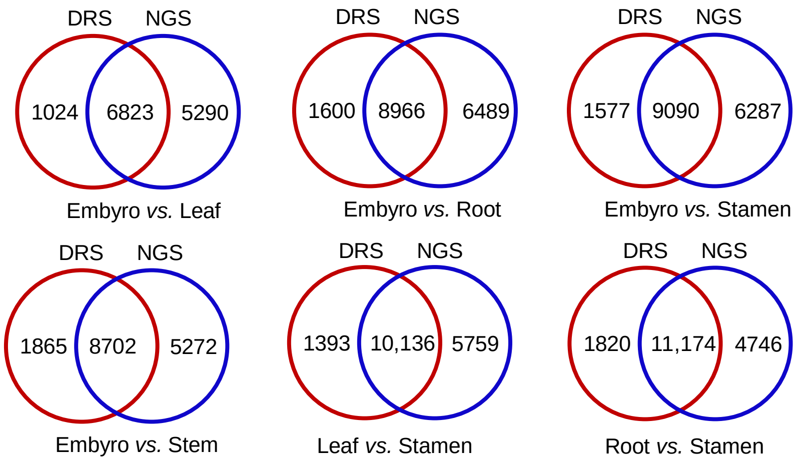
DRS
NGS
1024
6823
5290
Embyro vs. Leaf
DRS
NGS
1600
8966
6489
Embyro vs. Root
DRS
NGS
1577
9090
6287
Embyro vs. Stamen
DRS
NGS
1865
8702
5272
Embyro vs. Stem
DRS
NGS
1393
10,136
5759
Leaf vs. Stamen
DRS
NGS
1820
11,174
4746
Root vs. Stamen
